# DARPins detect the formation of hetero-tetramers of p63 and p73 in epithelial tissues and in squamous cell carcinoma

**DOI:** 10.1038/s41419-023-06213-0

**Published:** 2023-10-12

**Authors:** Alexander Strubel, Philipp Münick, Oliver Hartmann, Apirat Chaikuad, Birgit Dreier, Jonas V. Schaefer, Jakob Gebel, Christian Osterburg, Marcel Tuppi, Birgit Schäfer, Viktoria Buck, Mathias Rosenfeldt, Stefan Knapp, Andreas Plückthun, Markus E. Diefenbacher, Volker Dötsch

**Affiliations:** 1 Institute of Biophysical Chemistry and Center for Biomolecular Magnetic Resonance, Goethe University, 60438 Frankfurt, Germany; 2 Department of Biochemistry and Molecular Biology I, University of Würzburg, 97074 Würzburg, Germany; 3 Institute of Pharmaceutical Chemistry, Goethe University, 60438 Frankfurt, Germany; 4 Structural Genomics Consortium, Goethe University, 60438 Frankfurt, Germany; 5 Department of Biochemistry, University of Zurich, 8057 Zurich, Switzerland; 6 Department of Pathology, University of Würzburg, 97074 Würzburg, Germany

**Keywords:** Proteins, Skin cancer

## Abstract

The two p53 homologues p63 and p73 regulate transcriptional programs in epithelial tissues and several cell types in these tissues express both proteins. All members of the p53 family form tetramers in their active state through a dedicated oligomerization domain that structurally assembles as a dimer of dimers. The oligomerization domain of p63 and p73 share a high sequence identity, but the p53 oligomerization domain is more divergent and it lacks a functionally important C-terminal helix present in the other two family members. Based on these structural differences, p53 does not hetero-oligomerize with p63 or p73. In contrast, p63 and p73 form hetero-oligomers of all possible stoichiometries, with the hetero-tetramer built from a p63 dimer and a p73 dimer being thermodynamically more stable than the two homo-tetramers. This predicts that in cells expressing both proteins a p63_2_/p73_2_ hetero-tetramer is formed. So far, the tools to investigate the biological function of this hetero-tetramer have been missing. Here we report the generation and characterization of Designed Ankyrin Repeat Proteins (DARPins) that bind with high affinity and selectivity to the p63_2_/p73_2_ hetero-tetramer. Using these DARPins we were able to confirm experimentally the existence of this hetero-tetramer in epithelial mouse and human tissues and show that its level increases in squamous cell carcinoma.

## Introduction

The protein family of the tumor suppressor p53 consists of three members p53, p63, and p73 [1–3]. Of these, p63 and p73 share a higher sequence identity than each of them with p53. While p53 mainly acts as a tumor suppressor in somatic cells [4], p73 and p63 are important in developmental processes. Inactivation of p63 results in the loss of stratified epithelial tissues [5, 6] while p73 is essential for the development of multiciliated cells as found in trachea [7, 8]. Using immuno-fluorescence stainings of mouse skin we and others have shown that p63 and p73 are co-expressed in certain cell types, e.g. in the basal proliferative layer and in the outer root sheath of hair follicles [9–11] as well as the hair bulb, and the sebaceous gland in human and murine skin [10]. The laboratory of Jennifer Pietenpol further demonstrated that the epithelial tissue lining of the upper airways not only expresses p73 but also p63 [7].

**Fig. 1:**
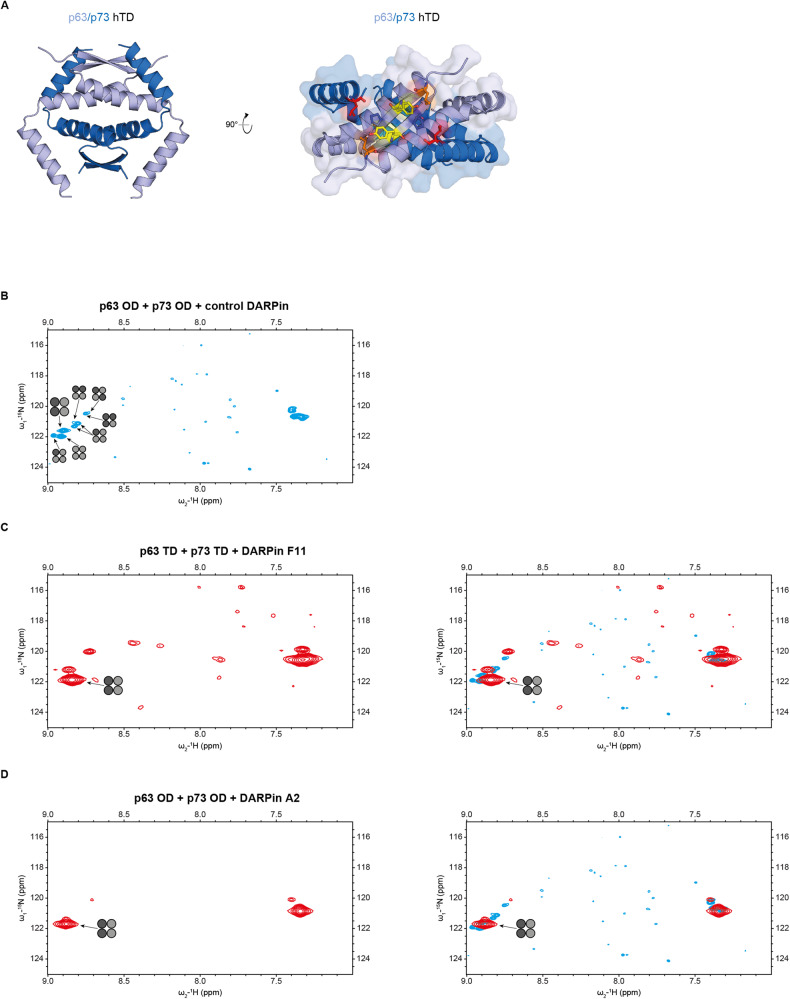
DARPins bind conformation-selective to the p63/p73 hetero-tetramer. Structure of the hetero-tetramer assembled by the tetramerization domains of p63 and p73. **A** The hetero-tetramer of p63/p73 (PDB 2NB1) is composed of two chains of each protein. For clarity reasons, the flexible C-termini of the p63 chains C-terminally of Q409 have been omitted. p63 chains (light blue) and p73 chains (dark blue) each form an independent dimer consisting of an N-terminal antiparallel β-sheet and one subsequent helix per monomer. These helices interact with the corresponding helices from the other dimer, thus forming the central tetramerization interface. Additionally, the C-terminal second helices reach across the tetramerization interface. The C-terminus of the second helix of p73 interacts with the β-sheet of p63, leading to the enhanced stability. The right image shows a view of the p63 β-sheet as a surface model with the second helices of p73 shown as explicit secondary structure element. The hydrophobic outward facing amino acids of the p63 chains are depicted as colored sticks (L361 in orange, Y363 in yellow, P365 in blue). The side chain of L396 of p73 (red, shown as sticks) penetrates into a hydrophobic pocket formed mainly by P365 and L361 of p63. **B** NMR spectrum of the selectively ^15^N-Lys labeled p63_2_/p73_2_ hetero-tetramer in the presence of the non-binding control DARPin. Only p73 was labeled, resulting in signals for the two lysine resonances K370 and K372. The signal of K372 in the [^15^N, ^1^H]-TROSY spectrum splits after mixing with the unlabeled p63 OD into eight individual signals that correspond to all stoichiometric and conformational possibilities for mixed p63/p73 tetramers. The individual species are marked with tetramer symbols in which light gray symbolizes p63 and dark gray p73. The signal of the p63_2_/p73_2_ hetero-tetramer is labeled with a larger symbol. **C** Addition of DARPin F11 reduces the complexity with only the p63_2_/p73_2_ hetero-tetramer being strongly populated. A few small peaks representing additional species with a population of less than 20% are visible. The weak resonances spread across the spectrum represent natural abundance peaks of the DARPin. On the right, an overlay of the spectrum on the left and the spectrum shown in (**B**) is provided. The larger line width of the DARPin - p63_2_/p73_2_ hetero-tetramer complex is due to the larger molecular weight of this complex. **D** The same as in (**C**) but with DARPin A2. This DARPin shows an even higher selectivity for the p63_2_/p73_2_ hetero-tetramer with additional resonances representing less than 10% of the population.

The high sequence identity between p63 and p73 has raised the question of whether both proteins can also directly interact with each other. This speculation was fueled by the fact that both proteins form tetramers via a dedicated and highly similar oligomerization domain (OD) that structurally constitutes a dimer of dimers. In in vitro experiments with the isolated ODs of p63 and p73 we and others were able to show that p63 and p73 form mixed tetramers, but both did not interact with the p53 OD [9, 12]. Surprisingly, these experiments further demonstrated that the thermodynamically most stable form within a mixture of p63 and p73 ODs is a tetramer consisting of a p63 dimer and a p73 dimer. To investigate the structural basis of this surprising effect we determined the NMR structure of this p63_2_/p73_2_ hetero-tetramer [11]. This structure revealed that hydrophobic interactions between the C-terminus of p73 with residues of the N-terminus of p63 exist (Fig. 1A) that cannot occur in either homo-tetramer and therefore explain the enhanced stability of the hetero-tetramer [11].

Further support for the physiological relevance of the hetero-tetramer came from cell culture experiments. In keratinocytes calcium treatment induces the expression of p73 while p63 is expressed already without calcium treatment. Upon p73 expression, we used co-immunoprecipitation experiments to demonstrate that p63 and p73 interact with each other in these cells [11]. In addition, ChIPseq analysis of p63 and p73 in ME180 cells demonstrated a large overlap of DNA binding sites of both proteins [13, 14]. This is not surprising as both proteins show a high sequence identity within their DNA binding domains [2], consistent with largely overlapping DNA sequence preferences. The results of sequential chromatin immunoprecipitation experiments with p63- and p73-specific antibodies, however, suggested that p63 and p73 indeed form hetero-tetramers [14]. Similarly, ChIPseq experiments in primary human keratinocytes, HaCaT and HCC1806 cells, identified overlapping p63 and p73 binding sites in the vicinity of a set of 38 genes involved in skin development, proliferation and wound healing [10], processes that both proteins are involved in.

All these results in combination with our biochemical and structural characterization of the preferred p63/p73 hetero-tetramerization make it likely that hetero-tetramers consisting of a p63 dimer and a p73 dimer exist in epithelial cells. Both p63 and p73 are expressed in multiple isoforms that differ in the presence or absence of the N-terminal transactivation domain as well as in C-terminal variations [15, 16]. While the predominant isoform of p63 is ΔNp63α, an isoform lacking high transcriptional activity on most promoters [15] and that is mainly involved in establishing an epithelial-specific chromatin landscape [17], several p73 isoforms differing in their transcriptional activity were found to be co-expressed in cells along with ΔNp63α [7, 8, 10]. This suggests that hetero-tetramers could be important for fine-tuning transcriptional programs.

Unfortunately, tools to investigate specific biological functions of the p63_2_/p73_2_ hetero-tetramer for visualization in cells or to use in hetero-tetramer-specific ChIPseq experiments are currently not available. We have, therefore, created Designed Ankyrin Repeat Proteins (DARPins) that selectively bind to such a hetero-tetramer. Here we show that they selectively bind to the hetero-tetramer with high affinity and thereby demonstrate that this hetero-tetramer is present in mouse and in human tissues.

## Results

Recently we have developed and characterized a set of DARPins as tight binders against all folded domains of p63 [18]. DARPins are small (14–18) kDa proteins consisting of multiple stacked helical hairpin units in which certain positions can be randomized to select tight binders for a folded target domain [19–22]. The advantage of DARPins relative to other selective binders such as antibodies is the absence of stabilizing disulfide bonds, making it possible to use these DARPins not only for detection in fixed cells but also for detection/inhibition in live cells. For the DARPin selection, we used the same p63_2_/p73_2_ hetero-tetramer construct of which we had determined the structure and that is based on rationally designed mutations in the p63 and the p73 ODs that enforce the formation of hetero-tetramers (by inhibiting the formation of homo-tetramers) [11]. The combination of an E363K mutation in the p73 OD and a K377E mutation in the p63 OD thus selectively stabilizes the p63_2_/p73_2_ hetero-tetramer [11]. We obtained several promising candidates from the in vitro DARPin selection by ribosome display that were cross-validated against the p63 and p73 homo-tetramers and we have characterized them further.

### Screening for binders of the p63_2_/p73_2_ hetero-tetramer

In previous NMR-based experiments of a mixture of the p63 and p73 ODs we had observed many different species that correspond to all possible stoichiometric and conformational combinations of both proteins within one tetramer [9]. The p63_2_/p73_2_ hetero-tetramer in these mixtures was the most abundant species, showing that it is the thermodynamically most stable form. As the DARPins had been selected against the stabilized p63_2_/p73_2_ hetero-tetramer our aim was to identify in an initial NMR screen those ones that clearly select the wild-type p63_2_/p73_2_ hetero-tetramer out of a mixture of different homo- and hetero-oligomers for further characterization. To reduce the signal overlap and simplify the spectra, the p73 OD was labeled selectively with ^15^N-lysine, resulting in two signals corresponding to Lys 370 and Lys 372. After mixing the p73 OD with unlabeled p63 OD, the Lys 372 signal split into eight individual signals corresponding to all possible combinations as reported previously [9, 12] (Fig. 1B). Adding a control DARPin did not change the spectrum but addition of either DARPin F11 or A2 resulted in disappearance of almost all resonances with the exception of those of the p63_2_/p73_2_ hetero-tetramer (Fig. 1C, D). Some additional peaks of low signal intensity probably represent DARPin—hetero-complexes of differing stoichiometry but these oligomers represent less than ~20% of the p63_2_/p73_2_ heterodimer population in case of the DARPin F11 and less than 10% in case of the DARPin A2. Other DARPins showed far more complex spectra in this assay hinting at a less stringent stoichiometric selection or otherwise different binding modes (Supplementary Fig. 1). Due to this heterogeneity these DARPins were left out and only DARPins F11 and A2 were further characterized. Of these DARPin F11 represents a DARPin with three internal helical repeats and DARPin A2 a DARPin with only two internal helical repeats, always flanked by an N- and C-capping repeat.

### Affinity and selectivity of binding

**Fig. 2:**
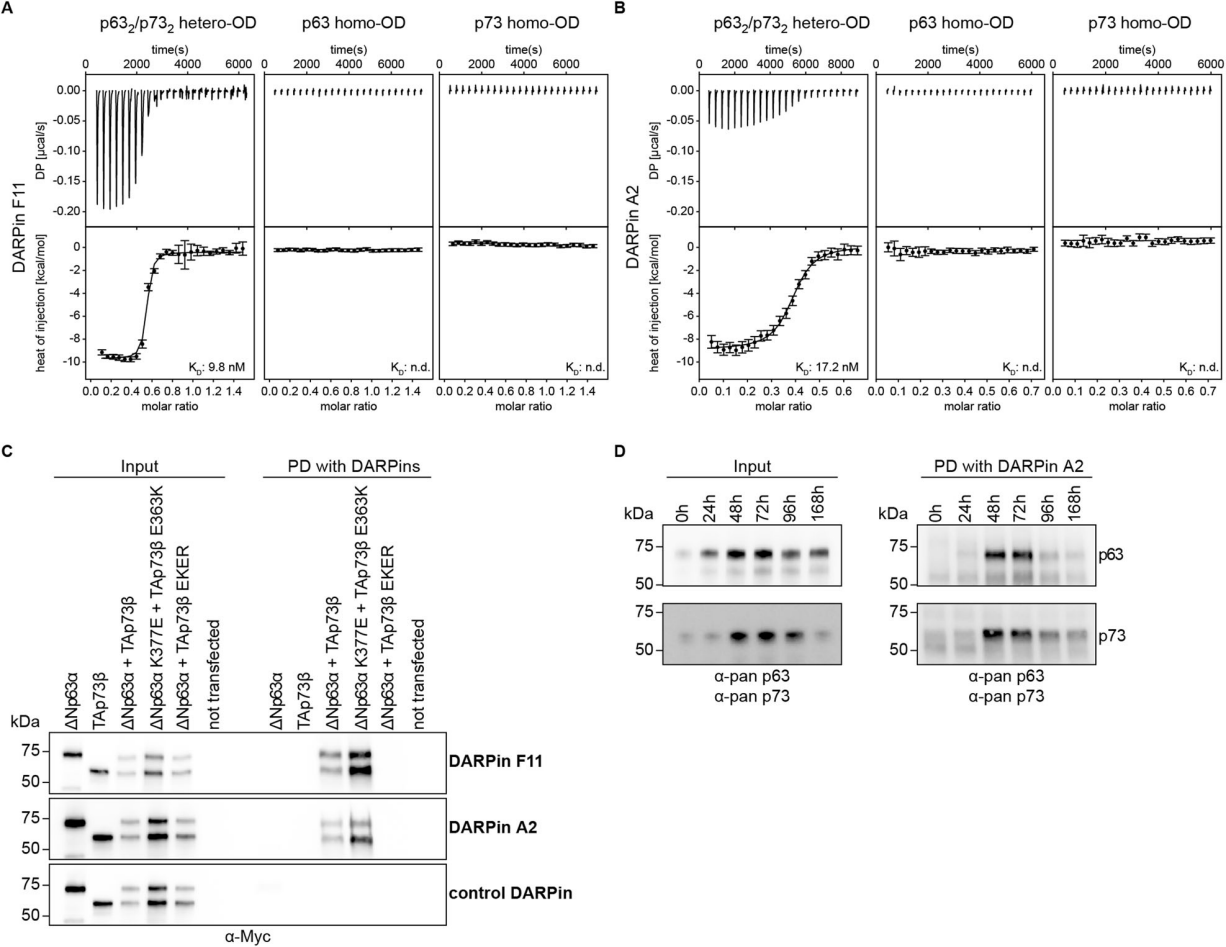
Interaction studies of the p63_2_/p73_2_ hetero-tetramer and the two homo-tetramers with the selected DARPins using Isothermal Titration Calorimetry (ITC). The experiments show a specific and very high-affinity binding of the DARPin to only the p63_2_/p73_2_ hetero-tetramer. **A** ITC data for DARPin F11. **B** ITC data for DARPin A2. The top diagram displays the raw measurement and the bottom diagram shows the integrated heat per titration step. The K_D_ value for the interaction is given. Thermodynamic parameters are provided in Supplementary Fig. 2B. **C** Pulldown experiments with the DARPins F11, A2 and a control DARPin. H1299 cells were transiently transfected with the indicated expression plasmids. The input signals are shown on the left, the signals after pulldown on the right. The p73 E363K and p63 K377E mutants enforce hetero-tetramer formation, the p73 E363R, K370E, E373R and R390D (EKER) mutant prevents it. **D** Pulldown experiments with the DARPin F11 and HaCaT cells. The left side shows the input signals, the right side the signals following pull-down experiments with the DARPin. The upper panel represents Western blot results with an anti-p63 antibody, the lower panel the corresponding results with an anti-p73 antibody. The time-line indicates the time following treatment with calcium, which results in a transient spike of p73 expression.

For the selected DARPins we measured the binding affinity using isothermal titration calorimetry (ITC). The bacterially expressed p63 (amino acids 358–416, K377E) and p73 (amino acids 351–398, E363K) ODs were mixed and purified via size exclusion chromatography showing that they form a hetero-tetramer, as expected [11]. Titration with the DARPins yielded dissociation constants of *K*_D_ = 10 nM for DARPin F11 and *K*_D_ = 17 nM for DARPin A2 and a 2:1 (DARPin : hetero-tetramer) stoichiometry, while a control DARPin did not bind at all (Fig. 2A, B, Supplementary Fig. 2A, B). We also measured binding to the two homo-tetrameric ODs of p63 and p73 by ITC but could not detect any binding, demonstrating that the interaction of both DARPins with the hetero-tetramer is not only highly affine but also highly specific.

Next, we tested if the DARPins would detect selectively hetero-tetramers of the full-length proteins in a cell culture lysate. For this purpose, we overexpressed ΔNp63α and TAp73β in H1299 cells. Cell lysates were incubated with the DARPins containing a FLAG-tag immobilized to an anti-FLAG antibody, itself immobilized on protein G magnetic beads. As a negative control, we used a E363R, K370E, E373R and R390D mutant of TAp73β (EKER) that does not interact with the p63 OD and can thus only form homo-tetramers. As a positive control the p73 E363K and p63 K377E mutants that form only hetero-tetramers [11] were used. The results demonstrated that binding occurs only in the mixture of ΔNp63α and TAp73 and in the mixture of the mutants designed to enforce hetero-tetramer formation (Fig. 2C, Supplementary Fig. 5). No signal was detected in cell lysates expressing only ΔNp63α or TAp73β or a mixture of ΔNp63α and the EKER mutant of TAp73β (that prevents hetero-tetramer formation). A control DARPin did not show any pulldown signal, either. These results confirm the specificity of both DARPins and show that hetero-tetramers can be pulled down from cell lysate in the context of full-length proteins.

We investigated this selectivity further in the more relevant HaCaT keratinocyte cell line derived from adult human skin. These cells express ΔNp63α and can be triggered by adding calcium to express p73 with a peak in expression between 48 and 72 h following calcium treatment. This system can be used to simulate skin differentiation. p63 and p73 are co-expressed in the basal layer of the skin but the expression level of both proteins vanishes with increasing differentiation state during epidermal development with p73 being exclusively expressed in the basal layer and p63 also in the supra-basal layer [9, 15]. The p63 and p73 expression levels upon induction of differentiation were examined in a time-dependent manner (Fig. 2D, Supplementary Fig. 5) using specific antibodies. All time points were also subject to pulldown analysis using biotinylated DARPin A2 immobilized on streptavidin-coated magnetic beads to assess the formation of p63_2_/p73_2_ hetero-tetramers. During the expression time of the p73 peak a strong pulldown signal using the DARPin A2 was detected that was not present at times in the absence of p73 expression (Fig. 2D, Supplementary Fig. 5), while a control DARPin did not show binding at any time point. These results also confirm that the mutations introduced in the ODs of p63 and p73 to stabilize the p63_2_/p73_2_ hetero-tetramer for the DARPin selection are not necessary for detecting the hetero-tetramer. Similar results were found with the wild-type but overexpressed version of both proteins in H1299 cells (Fig. 2C, Supplementary Fig. 5).

### Structural characterization

**Fig. 3:**
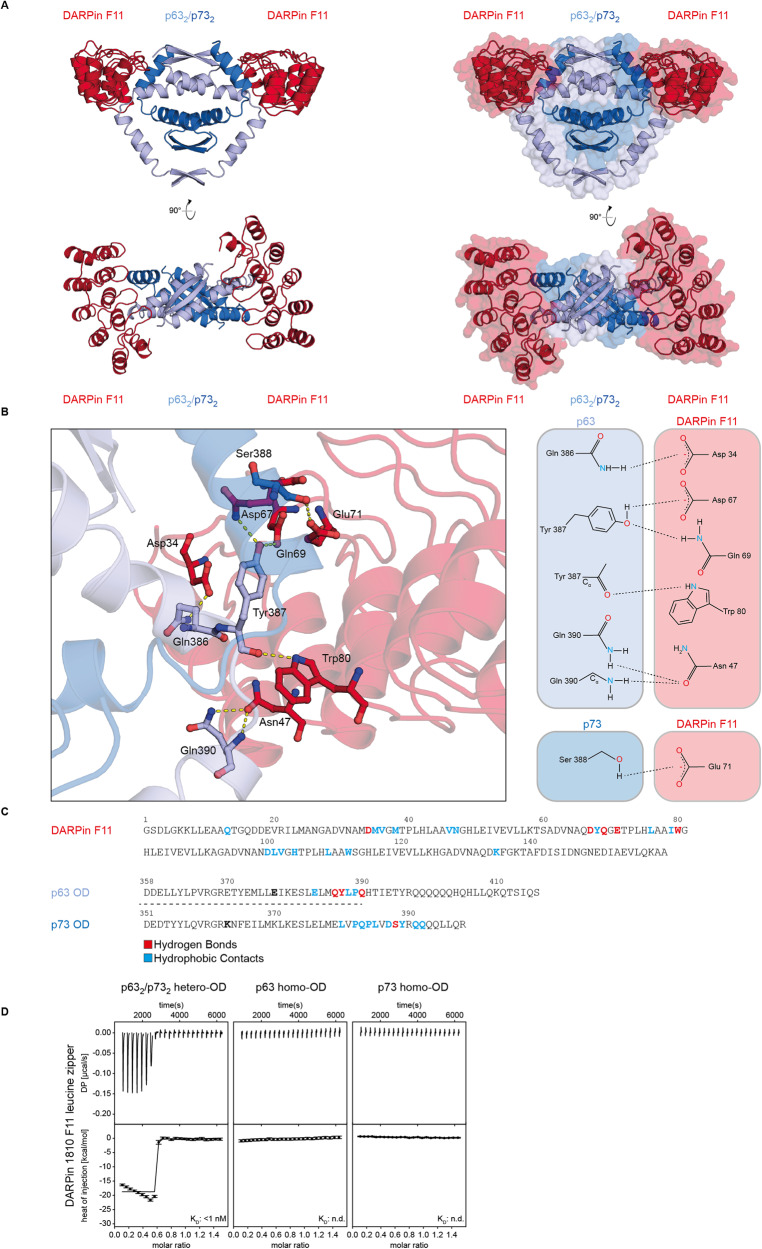
Crystal structure of the p63_2_/p73_2_ hetero-tetramer in complex with DARPin F11. **A** The structure is shown in different orientations rotated by 90°. The DARPin is depicted in red, the p63 dimer in light blue and the p73 dimer in dark blue. **B** Detailed analysis of the interface of DARPin F11 with the hetero-tetramer showing the interacting side chains of the complex crystal structure analyzed by LigPlot. **C** Sequence of the DARPin F11 and alignment of OD sequences of p63 and p73 with amino acids highlighted involved in the interaction with the DARPin. Hydrogen bond contacts are highlighted in red, hydrophobic contacts in blue. The two mutations, E363K in the p73 OD and K377E in the p63 OD, that were introduced to stabilize the p63_2_/p73_2_ hetero-tetramer are marked in bold. **D** ITC results of a titration of the p63_2_/p73_2_ hetero-tetramer with the leucine zipper dimerized DARPin F11. The functional affinity (avidity) is too high to be measured by direct titration. No binding can be observed for the p63 and p73 homo-ODs.

For a structural understanding of the interaction and the selectivity, we solved the X-ray crystal structures of complexes of both DARPins with the p63_2_/p73_2_ hetero-tetramer stabilized by mutations, thus the same DARPins that were used for the in vitro selection (Fig. 3A–C, Supplementary Fig. 3A–F and Supplementary Table 1). In addition, we solved the X-ray crystal structure of the DARPin F11 also in complex with the wild-type p63_2_/p73_2_ hetero-tetramer (Supplementary Fig. 3G). The structures of DARPin F11 with the mutant p63_2_/p73_2_ and the wild-type p63_2_/p73_2_ hetero-tetramers were indistinguishable, displaying the same intermolecular contacts. This, again demonstrates that the mutations that were necessary for the in vitro generation and selection of the DARPins (by holding the heterotretramer in a stable configuration) were not important for the binding of the DARPins. A detailed structural analysis further revealed that both DARPins bound to the hinge region between the first and the second helix of both p63 and p73 ODs. This second helix represents a special feature of both proteins that are not present in p53, and this explains why p63 and p73 interact strongly with each other but both do not interact with the p53 OD [9, 12]. The DARPins formed extensive hydrophobic contacts and hydrogen bonds with p63 and p73, which also explained the high selectivity relative to both homo-tetramers.

### Dimerization of DARPins further boost the affinity

The analysis of the DARPin–hetero-tetramer crystal structures revealed that the C-termini of both DARPins within the complex were pointing into the same direction, which enables the efficient dimerization of the DARPins via a leucine zipper for increasing the avidity. We, therefore, fused the DARPin F11 C-terminus via a (G_4_S)_4_ linker to the leucine zipper sequence of yeast GCN4 (amino acids 250–281) [23, 24]. Size exclusion chromatography showed a shift in the elution peak consistent with the formation of a dimer (Supplementary Fig. 3H). Using this dimer in ITC measurements with the p63_2_/p73_2_ hetero-tetramer (stabilized by mutations) revealed a very high, sub nM functional affinity that was too high to be accurately measured in a direct titration experiment but these data proved that the affinity of this bivalent DARPin construct can reach or exceed that of antibodies (Fig. 3D). No binding of the dimerized DARPin to the p63 and p73 homo-ODs were detected and a dimerized control DARPin also did not show binding (Supplementary Fig. 3I, J).

### Immuno-fluorescence detection of the p63_2_/p73_2_ hetero-tetramer

**Fig. 4:**
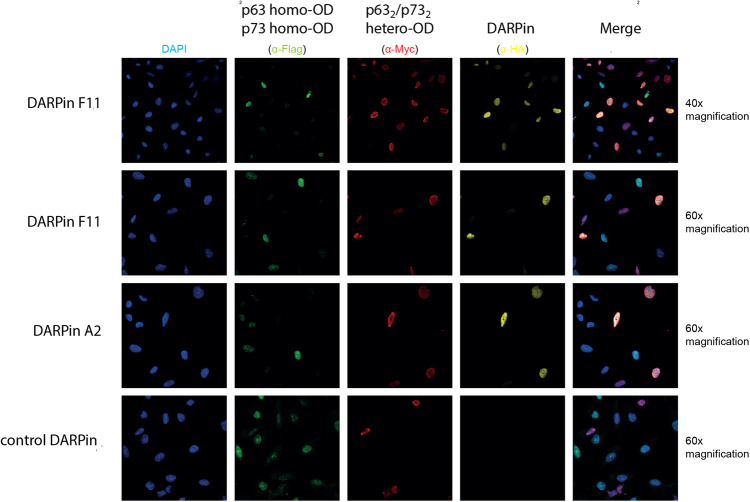
Testing the specificity of the DARPin F11 in staining experiments. Two U-2 OS-derived cell lines were mixed. One of them stably expressed both p63 and p73 mutants that exclusively form the p63_2_/p73_2_ hetero-tetramer (E363K in the p73 OD and K377E in the p63 OD). The other cell line expressed wild type p63 and a p73 mutant that allow only the formation of p73-homo-tetramers (E363R, K370E, E373R, and R390D). Cells expressing the p63_2_/p73_2_ hetero-tetramer can be identified by an anti-myc antibody (MYC-tag both on p63 and p73), cells expressing only the homo-tetramer forms by an anti-FLAG antibody (FLAG-tag on both p63 and p73). DARPins were visualized via an anti-HA-tag antibody. As can be seen, only cells positive for the anti-MYC antibody were also positive for the anti-HA-tag antibody staining, indicating specific binding by the DARPin with virtually no background staining. The last column shows a merged image of the staining results with the MYC-, FLAG- and HA-tag antibodies. A control DARPin (cDP) showed no staining. DAPI staining is provided in the first column. The upper row provides an image of the same samples shown directly below just with a lower magnification to display a larger area.

The results reported so far demonstrated that we can use the DARPins as tools for interaction studies by performing affinity-precipitation experiments. We also wanted to develop these DARPins into a tool for detecting the p63_2_/p73_2_ hetero-tetramer in cells and tissues. For this purpose, we modified the DARPin F11 with a C-terminal HA-tag. To evaluate the specificity of the staining we created two stable expressing U-2 OS cell lines that either express both hetero-specific or both homo-specific mutants of ΔNp63α and TAp73β. The homo versions of the p63 and p73 proteins were FLAG-tagged and the hetero versions of these proteins MYC-tagged. Both cell types were mixed, prepared using methanol fixation, treated with a DARPin solution and stained for the FLAG-tag (showing homo-tetramers), the MYC-tag (showing hetero-tetramers) and for the HA-tag (showing the DARPin). The results presented in Fig. 4 demonstrate that only cells stained with the anti-MYC antibody also showed an anti-HA tag signal, confirming that the DARPin bound very specifically and virtually without any background staining to the p63_2_/p73_2_ hetero-tetramer.

### In vivo detection of the p63_2_/p73_2_ hetero-tetramer

**Fig. 5:**
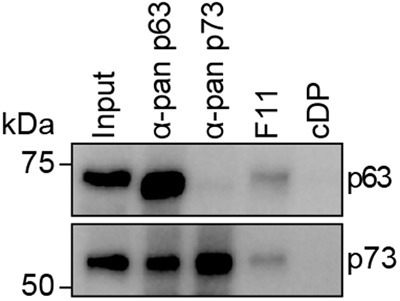
Pulldown experiments with primary mouse tissue. FLAG-tagged DARPin F11 or control DARPin (cDP) were immobilized on protein G magnetic beads saturated with anti-FLAG antibody and incubated with primary tissue lysates of mouse skin. As positive controls, pan-p63 and pan-p73 antibodies were immobilized on protein G magnetic beads and also incubated with primary tissue lysates from mouse skin. The pulldowns were analyzed by western blot using the same pan-p63 and pan-p73 antibodies as primary antibodies and a light chain specific secondary antibody (mouse anti-rabbit light chain) to prevent the detection of the heavy chain of the antibodies used for pulldown (as p63 has the same size as the heavy chain). The pulldown experiment was performed in biological triplicates with one exemplary replicate shown.

The characterization of the DARPins suggested so far that they are selective tools to further study the biological function of the p63_2_/p73_2_ hetero-tetramer. As a first step, we wanted to investigate the existence of this hetero-tetramer in primary tissue. In a first experiment, we wanted to test if we can use these DARPins to precipitate the hetero-tetramer from mouse skin. Tissue lysate of mouse skin was incubated with FLAG-tagged DARPin F11 immobilized on protein G magnetic beads saturated with anti-FLAG antibody. As positive controls pan-p63 and pan-p73 antibodies were immobilized on protein G magnetic beads and also incubated with mouse skin lysate. The results showed that DARPin F11 pulled down both p63 and p73 indicating the formation of p63/p73 hetero-tetramers in mouse skin (Fig. 5, Supplementary Fig. 5). The control DARPin showed no interaction. p63- and p73-specific antibodies immunoprecipitated their respective target but also showed co-IPs of the potential interaction partner (a strong co-IP signal for p73 in the p63-IP and a weak p63 signal in the p73-based IP). While these antibody-based co-IPs indicate the interaction between p63 and p73, they cannot prove the existence of a hetero-tetramer, as the interaction could also be mediated via other domains or even via other proteins. The positive pulldown with the DARPin F11, in contrast, demonstrated for the first time that a hetero-tetrameric complex exists in mouse skin.

**Fig. 6:**
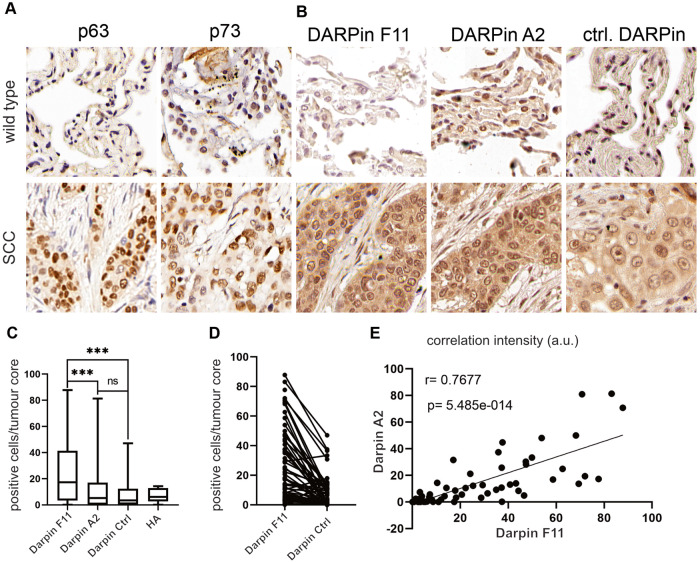
DARPins A2 and F11 detect the p63_2_/p73_2_ hetero-tetramer in human NSCLC-SCC. **A** Representative immunohistology stainings against endogenous p63 (clone 4A4) in matched non-transformed and human NSCLC-SCC. Scale bar = 1 mm or 2 mm, respectively. **B** Immunohistology analysis of non-transformed and matched NSCLC-SCC tumors exposed to HA-tagged control-DARPin and DARPins A2 and F11 as indicated, followed by HA-epitope specific antibodies. The tumor area is highlighted by dashed lines. Scale bar = 50 µm. **C** Quantitative analysis of IHC intensity in human SCC using DARPins F11 and A2, compared to control DARPin in mirco-array sections. The analysis was conducted with the image analysis software QuPath (0.4.2). **D** Parallel coordinate plots of total positive cells for DARPin F11 and control DARPin. Every connected dot represents a tumor sample of one patient with positive immunohistology signal in cells [%] of DARPin F11 or control DARPin. Mann-Whitney Test *p* > 0.001. **E** Pearson correlation between positive immunohistology signals of DARPin F11 versus DARPin A2 in human SCC tumor cores of a NSCLC tissue micro array (R = Pearson correlation coefficient, p = two tailed *t* test).

Pulldown experiments as described above work with lysed cells. Potentially the lysis during which the cellular content gets diluted and complexes (on the DNA) get potentially disrupted could be the reason that p63 and p73 could mix and form hetero-tetramers, while they would form only homo-tetramers in intact cells. To address the question whether hetero-tetramers exist in intact cells, we developed staining protocols for different tissues. First, we investigated the co-abundance of p63 and p73 in murine skin sections (Supplementary Fig. 4A, B). Here, hair follicles and the epidermis showed high abundance of both transcription factors, and the DARPins F11 and A2 showed positive reactivity towards double-positive cells, while the control DARPin only demonstrated low immunoreactivity (Supplementary Fig. 4A, B), thereby confirming that cells not only co-express p63 and p73 but also form the p63_2_/p73_2_ hetero-tetramer. As a tumor entity driven by p63 expression, next, we investigated the ability of DARPins to detect hetero-tetramers in human Non-Small Cell Lung cancer samples, especially Squamous Cell Carcinoma (SCC). To this end, tissue microarrays comprising patient-matched non-transformed and tumor tissue, were used (Fig. 6A). In non-transformed lung alveolar tissue, p73 was detectable in AT-2 cells, while, in line with previous reports, p63 failed to be detected in this tissue compartment. Consequently, the DARPins F11 and A2 were not detectable in control tissue sections, as seen by a signal intensity comparable to control tissue immunostainings using the unspecific control DARPin containing an HA-tag (Fig. 6B). In lung squamous cell carcinoma, p63 not only serves as a prognostic marker but also as an identity-defining oncoprotein [25] and was detected in tumor cells only (Fig. 6A). This tumor entity also showed increased abundance of p73, as seen by IHC staining (Fig. 6A). Here, DARPin F11 and A2 showed a high reactivity in cells positive for p63 and p73 (Fig. 6B). The use of digital pathology and image analysis tools demonstrated a significant increase in IHC intensity in human SCC for DARPins F11 and A2, when compared to control DARPin (Fig. 6C, D) and an overall high degree of IHC staining correlation of positive cells between DARPin F11 and A2 in p63_2_/p73_2_ double-positive cells (Fig. 6D, E). This is consistent with significant amounts of p63_2_/p73_2_ hetero-tetramer being present in these tumors. Comparable results were observed in a murine model of NSCLC-ADC and SCC, where oncogenesis was induced by CRISPR-mediated genome editing of somatic cells by introducing a *G12D* mutation in the endogenous locus of *Kras* (*K*), loss of function mutations in *Tp53* (*P*) and in the tumor suppressors *Fbxw7* (*F*) or *Stk11/Lkb1* (*L*), respectively, which had been published previously [26, 27]. The presence of both major NSCLC subtypes, ADC and SCC, was confirmed by IHC against the lineage markers TTF-1 (ADC+/SCC−), Krt5 (ADC−/SCC+) and p63 (ADC−/SCC+, Supplementary Fig. 4E–G). Here, only SCC tumors were positive for DARPin F11 and A2, while ADC failed to show a positive staining in the nucleus of tumor cells (Supplementary Fig. 4G, H). Overall our data demonstrate in vivo, that DARPins can be utilized to identify and label hetero-multimers comprising p63 and p73, in human lung cancer as well as in preclinical model systems.

## Discussion

The results presented here demonstrated for the first time that the p63_2_/p73_2_ hetero-tetramer exists in the skin and that this hetero-tetramer even becomes more abundant in human squamous cell carcinoma tissue. The DARPins that we have generated and characterized here provide an ideal tool for the further investigation of the biological function of the hetero-tetramer as well as its role in pathogenic conditions. What could be the biological function of the hetero-tetramer? The sequence preference of the p63 and p73 DNA binding domains is very similar and it is unlikely that the p63_2_/p73_2_ hetero-tetramer will significantly differ in this sequence preference. One model for a potential biological function of the hetero-tetramer takes the different isoforms of p63 and p73 into account that are expressed in epithelial tissues [15, 28, 29]. Both proteins can in principle form multiple different isoforms that are created by the combination of two different promoters, producing isoforms with either the full-length transactivation domain (TA-isoforms) or a truncated N-terminus lacking the transactivation domain (ΔN-isoforms) and multiple C-terminal splicing events [15, 16]. For p63, only ΔNp63 isoforms, lacking the transactivation domain, are expressed in skin and other epithelial tissues (mainly ΔNp63α). The main function of this isoform is that of a pioneering transcription factor that structures the chromatin landscape for other transcription factors [30], which is consistent with the high number of DNA binding sites identified by ChIPseq analysis [7, 13, 14, 30]. In the case of p73, however, multiple isoforms are expressed. In the basal layer of normal skin (stratified squamous epithelial tissue) ΔNp73α and ΔNp73β have been identified [10]. It is conceivable that in certain cells the rather structural role of ΔNp63α is switched to a more transcriptional one by the formation of hetero-tetramers with p73 isoforms. In particular, TAp73 isoforms would increase the transcriptional activity of such hetero-tetramers, but also the combination with ΔNp73α and with the transcriptionally more active ΔNp73β isoforms was shown to regulate the transcription of genes involved in epithelial maintenance, proliferation and wound healing [10]. In particular, with respect to wound healing, p73 seems to play a significant role. While the skin of p73^-/-^ mice does not show any morphological differences from skin of wild-type mice under normal conditions, wound healing seems to be delayed and less effective [10]. In wild-type mice, p73 expression increases in basal keratinocytes at the wound edge which explains the wound healing defects seen in p73^-/-^ mice. Here, the formation of complexes of ΔNp63α and p73 could be important for organizing transcriptional programs that participate in wound healing.

The importance of p73 is even more evident for the development of multiciliated cells (MCCs) in the pseudostratified epithelium of the trachea where inactivation of p73 results in loss of most cilia [7, 8]. Responsible for orchestrating a genetic program of MCC differentiation are TAp73 isoforms, while the C-terminal splice variants do not seem to be important [31]. The pseudostratified epithelium of trachea contains basal cells that are in contact with the basal membrane, neurodendocrine cells, club cells, mucin cells and multiciliated cells. These multiciliated cells express both p73 as well as the critical transcriptional regulator for MCC differentiation Foxj1, but no p63. In the basal compartment all cells are p63 positive and ~50% also express p73. Knockout of p63 results in a loss of almost all basal cells and a large increase in the population of multicilliated cells [7, 32]. Potentially, basal cells expressing both p63 and p73 might constitute a transition state between the basal cell population and the multicilliated cells that is driven by the hetero-tetramer.

Recently we have shown that the basal transcriptional activity of TAp63 is much higher than that of TAp73, and that the transactivation domains of both proteins are differently organized [33]: While p63 contains only a single, long transactivation domain that does not require further posttranslational modifications for high activity, the p73 transactivation domain is subdivided into two subdomains (similar to p53) and must be further activated by phosphorylation [33, 34]. Thus, the combination of hetero-tetramer formation including TAp73 isoforms, together with the activity of certain kinases, could constitute regulatory events for fine tuning the transcription of genes in cells expressing both p63 and p73.

## Materials and methods

### Selection and screening of DARPin binders specific for the p63_2_/p73_2_ hetero-tetramer

The selection and screening of DARPin binders specific for p53 family members was described before [18]. In brief, expression plasmids of p63 and p73 oligomerization domains carrying either the p63 K377E or the p73 E363K mutation (designed to form exclusively the p63_2_/p73_2_ hetero-tetramer) were cloned. The p63 OD was in addition N-terminally fused with an Avi-tag. *E. coli* BL21(DE3) Rosetta cells were transformed with both plasmids and a plasmid of *E. coli* biotin ligase BirA for protein production and in vivo biotinylation. Cells were grown in 2xYT medium supplemented with 10 µM biotin and proteins were purified as described below and in reference [18]. The p63_2_/p73_2_ hetero-tetramer was formed by mixing of respective p63 OD and p73 OD mutants in an equimolar amount and incubation overnight at 16 °C in IEX A buffer (50 mM Tris, pH 8.5). The mixture was loaded onto a HiTrap Q HP anion exchange column (Cytiva) pre-equilibrated with IEX A buffer and eluted applying a linear gradient of IEX B (50 mM Tris, pH 8.5, 1 M NaCl) for 60 min from 0–1 M NaCl with a flow rate of 3 ml/min. Fractions containing the p63_2_/p73_2_ hetero-tetramer were collected and applied onto a Superdex 75 10/300 column (Cytiva) using SEC buffer. Central peak fractions were collected, concentrated to the desired concentration and flash-frozen in liquid nitrogen prior to storage at −80 °C until use. The purity and molecular size of all purified proteins were monitored by SDS PAGE and LC-ESI-TOF-mass spectrometry.

The biotinylated p63_2_/p73_2_ hetero-tetramer was immobilized on either MyOne T1 streptavidin-coated beads (Thermo Fisher Scientific) or Sera-Mag neutravidin-coated beads (Cytiva), depending on the selection round. Ribosome display selections were performed as described [18, 22, 35] based on a fully synthetic library consisting of N3C-DARPins containing three randomized internal repeats between N- and C-capping repeats, using a mixture of non-randomized and randomized N-terminal and C-terminal capping repeats [20, 35, 36]. The DARPin selection was performed in total over four rounds with decreasing concentrations of biotinylated p63_2_/p73_2_ hetero-tetramer using a semi-automatic KingFisher Flex MTP96 well platform. The selection included an off-rate selection with non-biotinylated target protein in the third round as well as with less stringent conditions in the fourth round [18, 22, 37]. The final enriched pool of cDNA encoding for putative DARPin binders was cloned as fusion construct into a bacterial pQE30 derivative vector (Qiagen) with an N-terminal MRGS(H)8-tag and C-terminal FLAG-tag via unique BamHI x HindIII sites containing a T5 lac promoter and lacIq for expression control. After transformation of E. coli XL1-blue, 380 single DARPin clones selected to bind the p63_2_/p73_2_ hetero-tetramer were expressed in 96 well format and lysed by addition of 250 mM Tris-HCl pH 8.0, 250 mM NaCl, 50 mM MgCl_2_, 50 mg/ml lysozyme, 100 mg/ml n-octyl β-D-thioglucopyranoside. Bacterial crude extracts of single DARPin clones were subsequently used in a Homogeneous Time-resolved fluorescence (HTRF)-based screen to identify potential binders. Binding of the FLAG-tagged DARPins to streptavidin-immobilized biotinylated p63_2_/p73_2_ hetero-tetramer or p63 homo-tetramer and p73 homo-tetramer, respectively, was measured using FRET (donor: Streptavidin-Tb cryptate (610SATLB, Cisbio), acceptor: mAb anti-FLAG M2-d2 (61FG2DLB, Cisbio). Experiments were performed at room temperature in white 384-well Optiplate plates (PerkinElmer) using the Taglite assay buffer (Cisbio) at a final volume of 20 μl per well. FRET signals were recorded after an incubation time of 30 min using a Varioskan LUX Multimode Microplate (Thermo Scientific). HTRF ratios were obtained by dividing the acceptor signal (665 nm) by the donor signal (620 nm) and multiplying this value by 10'000 to derive the 665/620 ratio. The background signal was determined by using reagents in the absence of DARPins.

### Cell culture

The keratinocyte cell line HaCaT (CLS Cell Lines Service GmbH, CLS-number 300493) was cultured in DMEM medium (Gibco), containing Chelex (Sigma)-treated 10% FBS (Capricorn Scientific), 100 U/ml penicillin (Gibco), 100 µg/ml streptomycin (Gibco) and 0.03 mM CaCl_2_ at 37 °C and 5% CO_2_. For induction of differentiation, the medium was supplemented with 2.8 mM CaCl_2_. The non-small cell lung cancer cell line H1299 (ATCC, CRL-5803) was cultured in RPMI medium 1640 (Gibco), containing 10% FBS (Capricorn Scientific), 100 U/ml penicillin (Gibco) and 100 µg/ml streptomycin (Gibco) at 37 °C and 5% CO_2_. The HaCaT and H1299 cell lines were obtained from ATCC. The T-REx-U-2 OS cell line was cultured in DMEM medium (Gibco), containing 10% FBS (Capricorn Scientific), 4 µg/ml blasticidin (Gibco), 333 µg/ml Zeocin (Gibco), 100 U/ml penicillin (Gibco), 100 µg/ml streptomycin (Gibco) and 1 mM pyruvate (Gibco) at 37 °C and 5% CO_2_. The T-REx-U-2 OS cell line was a gift from Christian Behrends (Ludwig-Maximilians-University (LMU), Munich, Germany). All cell lines used in this study were routinely tested for mycoplasma contaminations. For recombinant protein expression, the H1299 culturing medium was exchanged with antibiotic-free medium and cells were transfected using Lipofectamine 2000 transfection reagent (Thermo Fisher Scientific) according to the manufacturer’s recommendations. The medium was exchanged 6 h after transfection to the standard H1299 culturing medium.

### Generation of U-2 OS cells stably expressing p63 and p73 mutants that can either form homo- or hetero-tetramers exclusively

For the generation of stable cell lines inducible expressing ΔNp63α and TAp73β mutants the Flp-In T-REx system (Thermo Fisher Scientific) for homologous recombination of the selected genes was used. T-REx-U-2 OS cells were cultured for two weeks and then transferred into six-well plates for transfection using the Lipofectamine 2000 transfection reagent (Thermo Fisher Scientific) according to the manufacturer’s recommendations. The cells were transfected with pcDNA5/FRT/TO (Thermo Fisher Scientific) containing the ΔNp63α and TAp73β mutants as well as pOG44 (Thermo Fisher Scientific) expressing the Flp recombinase according to the manufacturer’s recommendations. After transfection, the cells were cultured in DMEM medium containing 10% tetracycline-free FBS (Bio Cell) for two days and transferred into 15 cm dishes. One day after reseeding the medium was exchanged to Selection Medium with DMEM containing 10% tetracycline-free FBS, 4 µg/ml blasticidin, 200 µg/ml hygromycin (Thermo Fisher Scientific), 100 U/ml penicillin, 100 µg/ml streptomycin and 1 mM pyruvate, and the cells were cultured until a non-transfected control showed no longer viable cells (about 10–14 days). For monoclone selection, twelve single colonies of each cell line were isolated, cultured and the expression of target proteins was tested by adding 1 µg/mL tetracycline (Thermo Fischer Scientific) to the selection medium for 24 h followed by western blot analysis of the protein expression levels. The best three monoclones of each target protein were selected for further experiments.

### Molecular cloning

For recombinant protein expression (of all p63, p73, and DARPin constructs) in *E. coli* a pET15b vector was used (Novagen, Merck KGaA). PCR-generated inserts were introduced in pET-15b-His_10_-TEV, pET-15b-His_10_-TEV-Avi or pET-15b-His_10_-TEV-HA (N-terminal His_10_-tag followed by a tobacco etch virus (TEV) protease cleavage site (and Avi- or HA-tag)) by subcloning using *Bam*HI and *Xho*I restriction sites. For transient expression in mammalian cells, PCR-generated inserts were introduced in pcDNA3.1(+) Myc (Invitrogen, Thermo Fisher Scientific Inc) by subcloning PCR-generated inserts using *Bam*HI and *Xho*I.

For the cloning of the leucine zipper-DARPin constructs the protein-coding sequence, including the DARPin, fused at its C-terminus to a (G_4_S)_4_ linker, followed by the leucine zipper sequence (amino acids 250–281 of yeast GCN4) was synthesized (Eurofins). This construct was inserted into a pET-15b-His_10_-TEV vector using *BamHI* and *XbaI*.

### Protein expression and purification

*E. coli* BL21(DE3) Rosetta cells were transformed with individual expression plasmids for protein production. Cells were grown in 2xYT medium to an optical density of 0.8. Protein expression was induced with 0.6 mM IPTG for 16 h at 16 °C. Cells were harvested by centrifugation, resuspended in IMAC A buffer (50 mM Tris, pH 8, 400 mM NaCl) supplemented with RNAse (Sigma), DNAse (Sigma), lysozyme (Sigma), self-made protease inhibitors (Protease inhibitor cocktail (PIC) (100x): 250 mM AEBSF, 25 mM leupeptin, 25 mM bestatin, 0.75 mM aprotinin, 12.5 mM E-64 and 2.5 mM pepstatin A dissolved in 50% methanol at 4 °C; solvent was evaporated under vacuum; stored at −20 °C; before use the PIC pellet was resuspended in 1 ml Milli-Q H2O or buffer; all chemicals bought from Carl-Roth GmbH & Co. KG, Germany) and lysed by sonication. The lysate was cleared by centrifugation at 4 °C, the supernatant was supplemented with 30 mM imidazole and applied onto a pre-equilibrated immobilized metal ion affinity chromatography (IMAC) column (HiTrap IMAC Sepharose FF, Cytiva). Bound protein was washed with IMAC A buffer supplemented with 50 mM imidazole and eluted by a step gradient with IMAC A buffer supplemented with 300 mM imidazole. The eluted protein was then simultaneously dialyzed to IMAC A buffer and digested with TEV protease. TEV protease and undigested protein were separated by a reverse IMAC step. The purified proteins were further polished and buffer exchanged by size exclusion chromatography (SEC) with SEC buffer (50 mM Tris, pH 8, 150 mM NaCl, 0.5 mM TCEP) using a Superdex 75 10/300 column (Cytiva). Central peak fractions presenting the p63_2_/p73_2_ hetero-tetramer were pooled, concentrated to the desired concentration (Amicon Ultra Centrifugal Filters, Millipore) and flash-frozen in liquid nitrogen prior to storage at −80 °C until use. The p63_2_/p73_2_ hetero-tetramer was formed as described above with the only difference that for the structure determination and biophysical investigations no Avi-tagged proteins were expressed.

### Size exclusion chromatography (SEC)

Size exclusion chromatography (SEC) experiments were carried out using an Äkta purifier system (Cytiva) and a Superdex 75 10/300 column (Cytiva). The column was loaded with max. 10 mg protein with a flow rate of 0.5 ml/min at 4 °C using the indicated buffers.

### NMR characterization experiments

DARPins as well as the p63 OD and p73 OD were expressed and purified from *E. coli* as described before [11] and the His-tags were cleaved off. Proteins were applied to SEC using a Superdex 75 10/300 column in NMR buffer (20 mM K_2_HPO_4_, pH 7.8, 100 mM KCl, 0.5 mM EDTA). For NMR experiments the p73 OD was ^15^N-labeled on lysine residues as previously described [11]. Respective DARPins were mixed in a 2.4:1 molar ratio (assuming to result in a 2:1 DARPin:hetero-OD complex), using an equimolar mixture of p63 OD and ^15^N-labeled p73 OD in NMR buffer. [^15^N, ^1^H] BEST-TROSY spectra were measured at 25 °C on Bruker Avance spectrometers with proton frequencies of 600–950 Mhz.

### DARPin biotinylation

DARPins containing an N-terminal Avi-tag were in vitro enzymatically biotinylated using the *E. coli* biotin ligase BirA. BirA was subcloned into a pET-15b-GFP-His_8_-TEV *E. coli* expression vector (Novagen, Merck KGaA) and was expressed and purified as described before [18]. The in vitro biotinylation was performed by mixing GFP-BirA in a 1:50 molar ratio with the respective DARPin containing an Avi-Tag in SEC buffer supplemented with 10 mM ATP, 10 mM MgCl_2_, 0.5 mM biotin. The mixture was incubated for 16 h at 16 °C and subsequently separated using a Superdex 75 10/300 column (Cytiva) and SEC buffer. Biotinylated DARPin fractions were pooled and analyzed by LC-ESI-TOF-mass spectrometry. Only DARPins showing close to 100% labeling efficacy were used for experiments.

### Gel electrophoresis and western blotting

Samples for subsequent immunoblotting were mixed with NuPAGE LDS buffer (Thermo Fisher Scientific) supplemented with 0.5 mM DTT, denatured at 95 °C and applied to 15% Mini PROTEAN TGX Stain-Free Precast Protein gels (Bio Rad). Proteins were then transferred onto 0.45 µm PVDF membranes using the Trans-Blot Turbo Transfer System (Bio Rad) according to the manufacturer’s recommendations. Membranes were blocked using TBS-T with milk (TBS, 0.05% (v/v) Tween 20, 5% skim milk powder, Sigma-Aldrich) with gentle shaking for 1 h at RT. WB blocking buffer was exchanged to 10 ml primary antibody solution (list of antibodies and dilutions, see below) in WB blocking buffer and membranes were incubated with gentle shaking overnight at 4 °C. Membranes were washed three times each with TBS-T for 10 min with gentle shaking at RT. Thereafter, membranes were incubated in secondary antibody (see below) diluted in WB blocking buffer gentle shaking for 1 h at RT. Membranes were again washed three times and stored in TBS-T at 4 °C until detection. Chemiluminescence was detected using the ChemiDoc Imaging System (Bio Rad) and Amersham ECL Prime WB Detection Reagent (Cytiva) according to the manufacturer’s recommendations. In case a second primary antibody for detection was applied, the membrane was washed again three times with TBS-T to remove remaining substrate, blocked with WB blocking buffer and proceeded as described before. For detection, the WB membrane was cut to remove the chemiluminescence signal from the first round.

The following antibodies and dilutions were used for immunoblotting detection: anti-myc (1:2000, clone 4A6, Millipore), anti-p63 (1:2000, ab124762, Abcam), anti-p73 (1:2000, ab40658, Abcam), anti-vinculin (1:2000, clone 7F9, Santa Cruz), mouse anti-rabbit light chain HRP (1:2000, 211-032-171, Jackson ImmunoResearch Europe Ltd.), goat anti-mouse HRP (1:5000, A9917, Sigma Aldrich), anti-FLAG (clone M2, Sigma-Aldrich), anti-HA (A190-107A, Bethyl Laboratories Inc.) and goat anti-rabbit HRP (1:2000, Jackson ImmunoResearch Europe Ltd.).

### Pulldown assays

Recombinant target proteins (cloned into a pcDNA3 vector) were expressed in H1299 cells. Endogenous target proteins were pulled from differentiated HaCaT cells or from tissue samples of eight-day-old (P8) female CD 1 mice, purchased from Charles River Laboratories. Cell and tissue lysates for pulldown assays were generated as described before [18]. Magnetic Dynabeads MyOne Streptavidin T1 (Thermo Fisher Scientific) were pre-incubated with an excess of biotinylated DARPins in Pulldown (PD) wash buffer (50 mM Tris, pH 8, 150 mM NaCl 0.1% (v/v) Tween-20) while rotating for 2 h at 4 °C. The beads were subsequently washed three times with PD wash buffer and were resuspended in PD wash buffer with the same volume as before to maintain magnetic bead concentrations. For PD experiments, 10 µl DARPin loaded beads were mixed with freshly prepared cell lysate or tissue lysate which were adjusted to a total volume of 1000 µl PD wash buffer supplemented with 1x Complete Protease Inhibitor (Roche). The PD mix was incubated rotating overnight at 4 °C and subsequently washed five times with 1000 µl PD wash buffer and eluted with LDS buffer by boiling at 70 °C for 10 min. Samples were analyzed by western blot as described before. All pulldown experiments in this study were performed as biological triplicates if not stated otherwise.

### Isothermal titration calorimetry

All Isothermal titration calorimetry (ITC) experiments were performed using a MicroCal VP-ITC microcalorimeter (Malvern Instruments Ltd., UK) with 1.4 mL cell volume. DARPins and target proteins were dialyzed against ITC buffer (50 mM HEPES, pH 7.4, 150 mM NaCl, 0.5 mM TCEP). Target proteins were titrated to constant concentrations of DARPin in 25 steps à 10 µl and 250 s spacing time at 25 °C. The reference power was set to 25 µCal/s and a stirring speed of 307 rpm. Automated unbiased baseline calculation and curve integration was done with NITPIC [38]. Thermodynamic parameters and the equilibrium constant were calculated by SEDPHAT [39] assuming an AB (1:1) hetero association model. The first data point was always excluded from the analysis.

### Crystallization

Protein complexes consisting of DARPin and target were prepared in SEC buffer by mixing purified proteins in 1:2 molar ratio (OD:DARPin). The protein mix was incubated overnight at 16 °C and complexes were separated from unbound proteins by SEC in crystallization buffer (20 mM Tris, pH 7.8, 50 mM NaCl, 0.5 mM TCEP) using a Superdex 75 10/300 column. The very central peak fractions corresponding to the complex were combined and concentrated to a final complex concentration of ~300 μM. Prior to plate setup the protein purity was analyzed by SDS-PAGE and LC-ESI-TOF-mass spectrometry. Crystallization was performed using the sitting drop vapor diffusion method at 20 °C with conditions shown in Supplementary Table 1. Obtained crystals were mounted using mother liquor containing 22% glycerol as a cryo-protectant before being flash-frozen in liquid nitrogen. Diffraction data were collected at the Swiss Light Source and processed and scaled using XDS [40] and Aimless [41], respectively. All crystal structures were determined by molecular replacement using Phaser [42] with the published structure PDB ID 2NB1 as search model. Model rebuilding was performed using COOT [43] and REFMAC5 [44] for refinement. Crystal statistics are summarized in Supplementary Table 1. Structures are deposited in the PDB with the accession numbers 8P9C, 8P9D, and 8P9E.

### Immunofluorescence staining

Stable T-REx-U-2 OS cell lines expressing either the respective p63/p73 homo- or hetero-mutants were mixed in 1:1 ratio and seeded on cover slips (Carl Roth). Expression of the target proteins was induced as described before [18]. Twenty-four hours after induction cells were washed twice with PBS and fixed with ice-cold MeOH (Carl Roth) for 15 min at −20 °C. Fixed cells were washed three times with PBS for 5 min each at RT. Cells were blocked with blocking buffer (PBS supplemented with 1% BSA (Carl Roth) and 0.1% Triton X-100, Carl Roth) for 1 h at room temperature. Blocked cells were incubated with 50 nM HA-tagged DARPin over night at 4 °C. Cells were washed six times with PBS-T (PBS supplemented with 0.1% Triton X-100, Carl Roth) and incubated with a mixture of goat anti-HA (1:250, a190138a, Bethyl), mouse anti-myc (1:500, clone 4A6, Millipore) and rabbit anti-FLAG (1:500, Abcam ab1162) antibodies in blocking buffer for 4 h at room temperature. The cells were again washed six times with PBS-T and incubated with a mixture of Alexa Fluor 488 anti-rabbit (1:200, A31573, Life Technologies), Alexa Fluor 568 anti-goat (1:200, A11057, Life Technologies) and Alexa Fluor 647 anti-mouse (1:200, A31571, Life Technologies) antibodies in blocking buffer for 2 h at room temperature. Slides were washed six times with PBS-T and coverslips were mounted using Mowiol (Carl Roth) mounting medium which was supplemented with DAPI (Thermo Fisher Scientific). The detailed recipe of the mounting medium can be found at CSH protocols (http://cshprotocols.cshlp.org/content/2006/1/pdb.rec10255). The slides were dried for several days before imaging with a LSM 780 confocal laser scanning microscope (Zeiss).

### Formalin-fixed and paraffin-embedded tissue

Tumor-bearing lungs were dissected and fixed in 10% NBF for 24 h. Lobes were separated and placed into an embedding cask. The tissue was processed in the tissue processor Thermo MICROM STP120 overnight with the following steps: 2 ×70% EtOH, 2 ×90% EtOH, 2 ×95% EtOH, 2 ×100% EtOH, 3 × Xylol and pre-heated paraffin.

Human samples were obtained from the Institute of Pathology, University Würzburg after informed consent was given. Paraffin-embedded sections of human and murine samples were cut into 2 sections with a microtome (Leica). The slides were de-paraffinized and rehydrated using the following protocol: 3 × 5 min in xylene, 2 × 3 min in EtOH (100%), 2 × 3 min in EtOH (95%), 2 × 3 min in EtOH (70%), 2 min in EtOH (50%) and 3 min in H_2_O. After de-paraffinization and rehydration, antigen retrieval was performed with 10 mM sodium citrate buffer (pH 6.0) in a microwave oven with three incubation times of each 5 min at 800 W, 650 W and finally 360 W. The samples were permeabilized with TBS, 0.1% Tween-20 for 10 min and washed with TBS and blocked for 1 h at room temperature with 10% goat serum, 1.5% BSA in TBS. The respective primary antibodies (anti-p63: ab124762, Abcam, anti-p73: ab40658, Abcam) were diluted 1:100 in blocking solution and incubated overnight at 4 °C. Next, the endogenous peroxidase was blocked with TBS containing 3% H_2_O_2_ for 10 min. Slides were developed with the SuperBoost™ HRP coupled secondary antibodies and with DAB (3,3’-diaminobenzidine) staining solution (SignalStainR DAB Substrate Kit, Cell Signaling 8059S) and counterstained with hematoxylin (Sigma H3136). Slides were scanned at 40× resolution using a Panoramic DESK II slide scanner (3D Histech) or Olympus VS120 and analyzed using QuPath (version 0.4.2) (https://qupath.github.io/).

For the DARPins, the slides were incubated with 1 µM HA-tagged DARPin in blocking solution overnight at 4 °C. After peroxidase block and washing with TBS, the slides were incubated with anti-HA-antibody (1:100) in blocking solution for 2 h at room temperature. The development was carried out as described above.

**Table:** 

SignalStainR DAB Substrate Kit	Cell Signaling	8059S
SuperBoost™ Goat anti-Mouse Poly HRP	Thermo Fisher Scientific	B40961
SuperBoost™ Goat anti-Rabbit Poly HRP	Thermo Fisher Scientific	B40962
Monoclonal mouse anti-HA (16B12)	Abcam	ab130275
Hematoxylin	Sigma	GHS332-1L

### Human lung cancer tissue microarrays (TMA)

TMAs were prepared as previously described [45]. In brief, paraffin molds were cast using an Arraymold Kit (IHC World, Kit D, IW-115, core diameter 2 mm, 36 cores). Human samples were cut and stained using haematoxylin and eosin and digitalized using a 3D Histech slide scanner (panoramic FLASH). Tumor and non-transformed tissues were identified, manually ‘punched’ and transferred. Upon completion, 3-µm thick sections were cut using a microtome and processed.

### Animal experiments

The mouse strain used was B6(C)-Gt(ROSA)26Sorem1.1(CAG-cas9*,-EGFP)Rsky/J (Jackson Laboratories, stock #028555). All animals were housed in standard cages in a pathogen-free facility on a 12 h light/dark cycle with ad libitum access to food and water. Animal health monitoring via sentinel animal screening is carried out in accordance with FELASA 2014 guidelines and conducted every 3 months. Adult mice (7–8 weeks old) were anaesthetized with isoflurane and intratracheally intubated with 60 µl AAV (1 × 10^11^ PFU) diluted in PBS. Viruses were quantified using Coomassie staining protocol [46] and via qPCR (https://www.addgene.org/protocols/aav-titration-qpcr-using-sybr-green-technology/). At the indicated time point, animals were sacrificed by cervical dislocation and lungs were dissected and fixed using 4% neutral buffered formalin (Sigma). Tumor burden was determined by calculating the percent tumor area relative to the total lung area for each animal using QuPath 0.4.2 (https://qupath.github.io/).

### Statistical analysis

Statistical analysis was performed using GraphPad Prism 9 (Graphpad Software Inc.).

### Supplementary information

Supplementary figures 1-4 and Supplementary Table 1

Original Data File

aj-checklist

8P9C validation report

8P9D validation report

8P9E validation report

## Data Availability

All data needed to be evaluated and the conclusions in the paper are present in the paper and/or the Supplementary Materials. PDB accession codes for the three crystal structures are 8P9C, 8P9D, and 8P9E.
