# Supplementary material for: DARPins detect the formation of hetero-tetramers of p63 and p73 in epithelial tissues and in squamous cell carcinoma
Source: Cell Death Dis. 2023 Oct 12;14(10):674. doi: 10.1038/s41419-023-06213-0 (PMC10570377; doi:10.1038/s41419-023-06213-0)
Supplement: Supplementary file 1 — Supplementary figures 1-4 and Supplementary Table 1 [file 41419_2023_6213_MOESM1_ESM.docx]

**Supplementary Material**

**
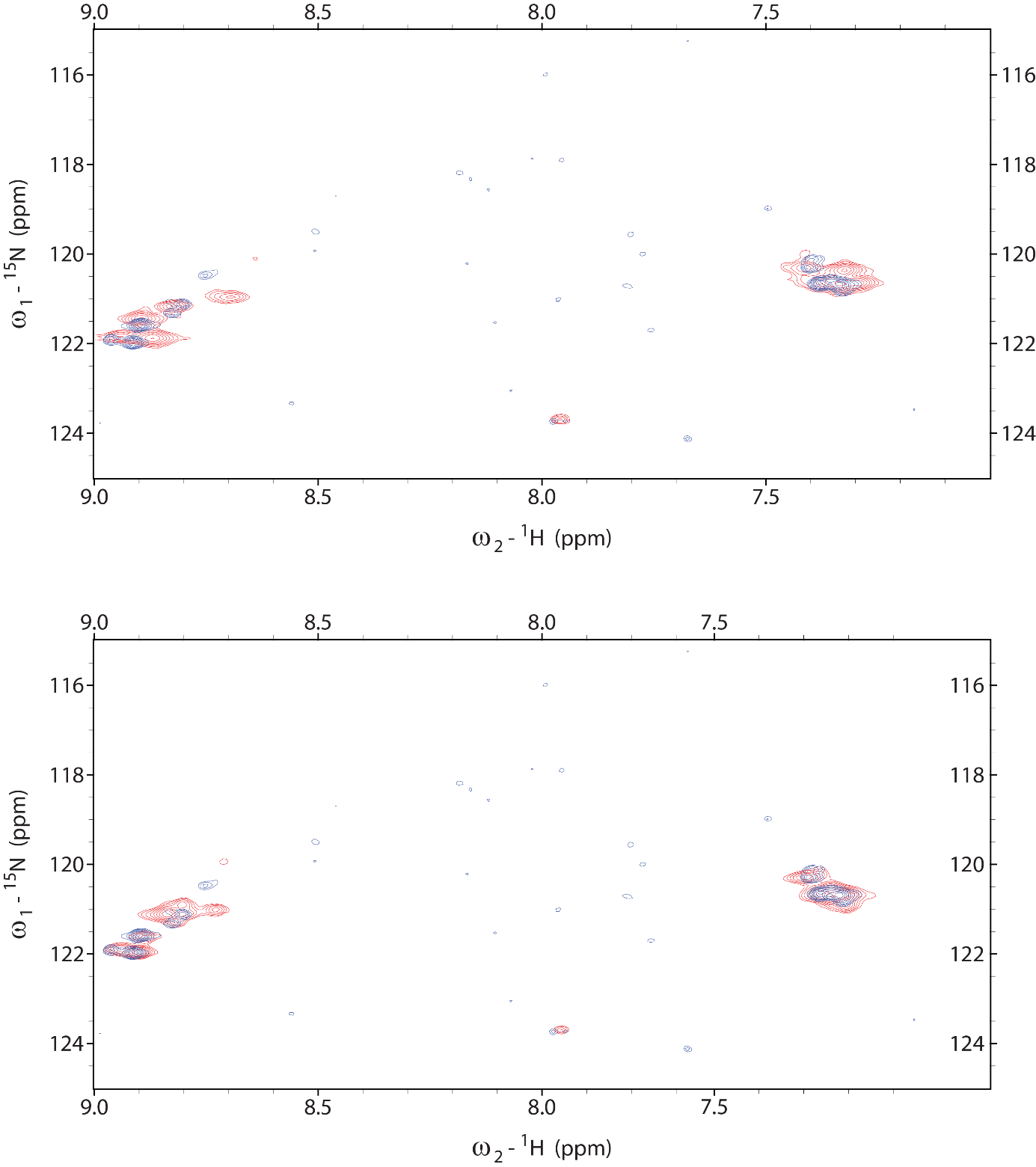
**

**Supplementary Fig. 1**

Examples of the overlay of NMR spectra of the selectively ^15^N-Lys labeled p63_2_/p73_2_ hetero-tetramer with control DARPin shown in blue and DARPin C4 (top) or DARPin D10 (bottom) shown in red. Only p73 was labeled, resulting in signals for the two lysine resonances K370 and K372. The signal of K372 in the [^15^N, ^1^H]-TROSY spectrum splits after mixing with the unlabeled p63 OD into eight individual signals that correspond to all stoichiometric and conformational possibilities for mixed p63/p73 tetramers. Addition of the DARPins C4 and D10 does not result in the selection of a single dominating species, demonstrating less conformational selectivity compared to the DARPins F11 and A2 that were further characterized.

**
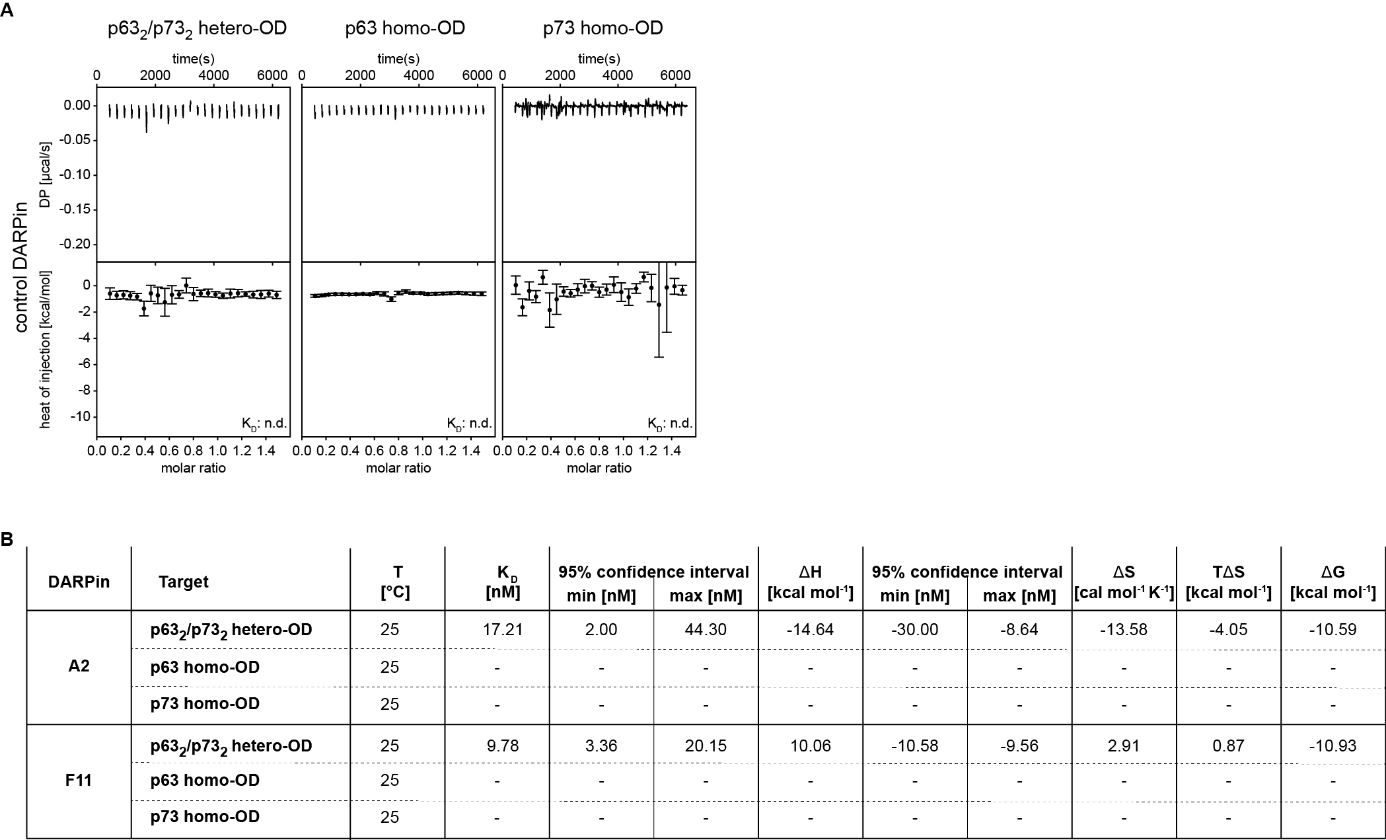
**

**Supplementary Fig. 2**

(**A**) Results of ITC experiments with the control DARPin. This control DARPin does not interact with the stabilized p63_2_/p73_2_ hetero-tetramer, the p63 homo-OD or the p73 homo-OD. The top diagram displays the raw measurement and the bottom diagram shows the integrated heat per titration step.

(**B**) Summary of the thermodynamic parameters of the interaction of DARPins A2 and F11 with the stabilized p63_2_/p73_2_ hetero-tetramer, the p63 homo-OD and the p73 homo-OD obtained by ITC measurements.


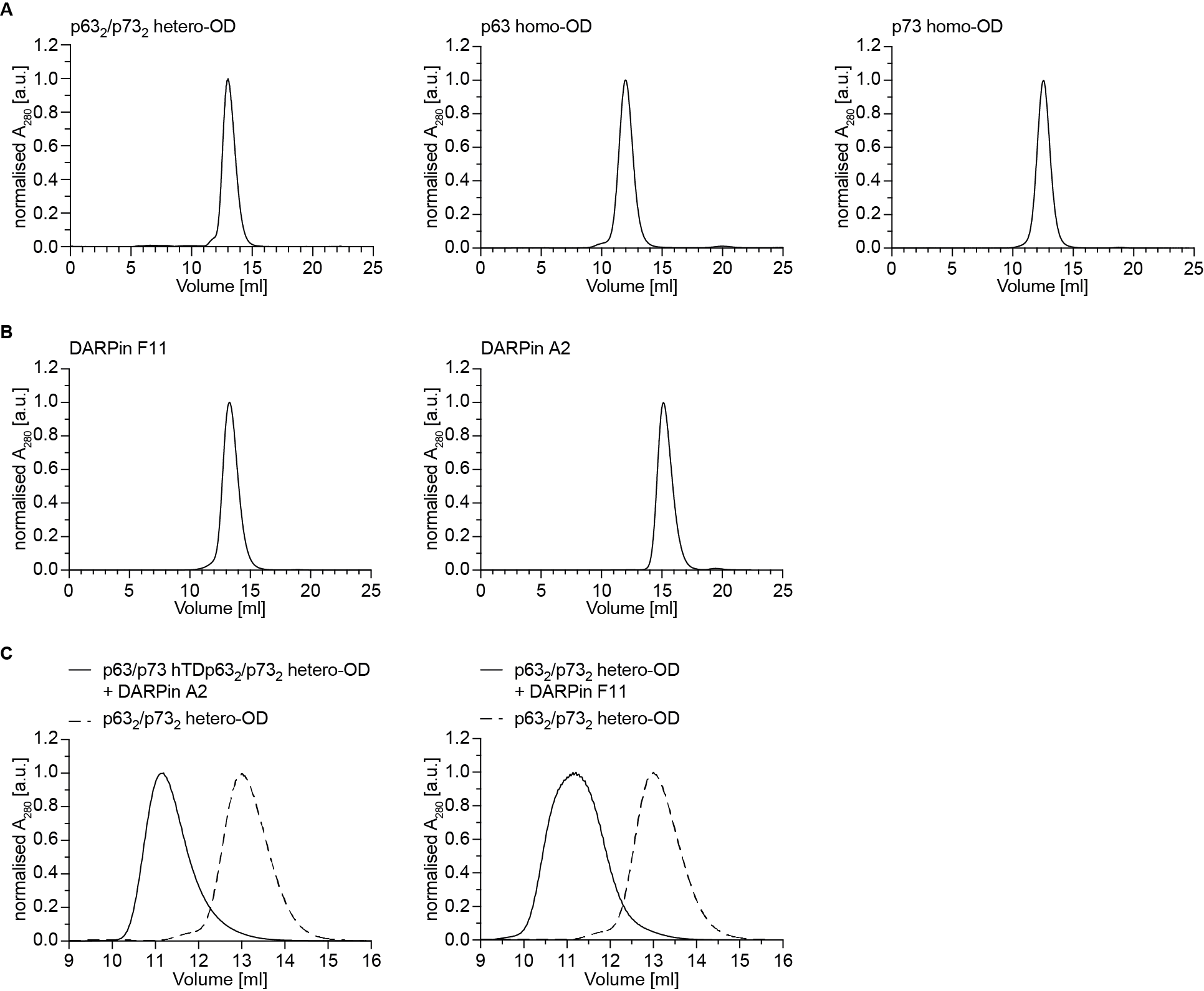


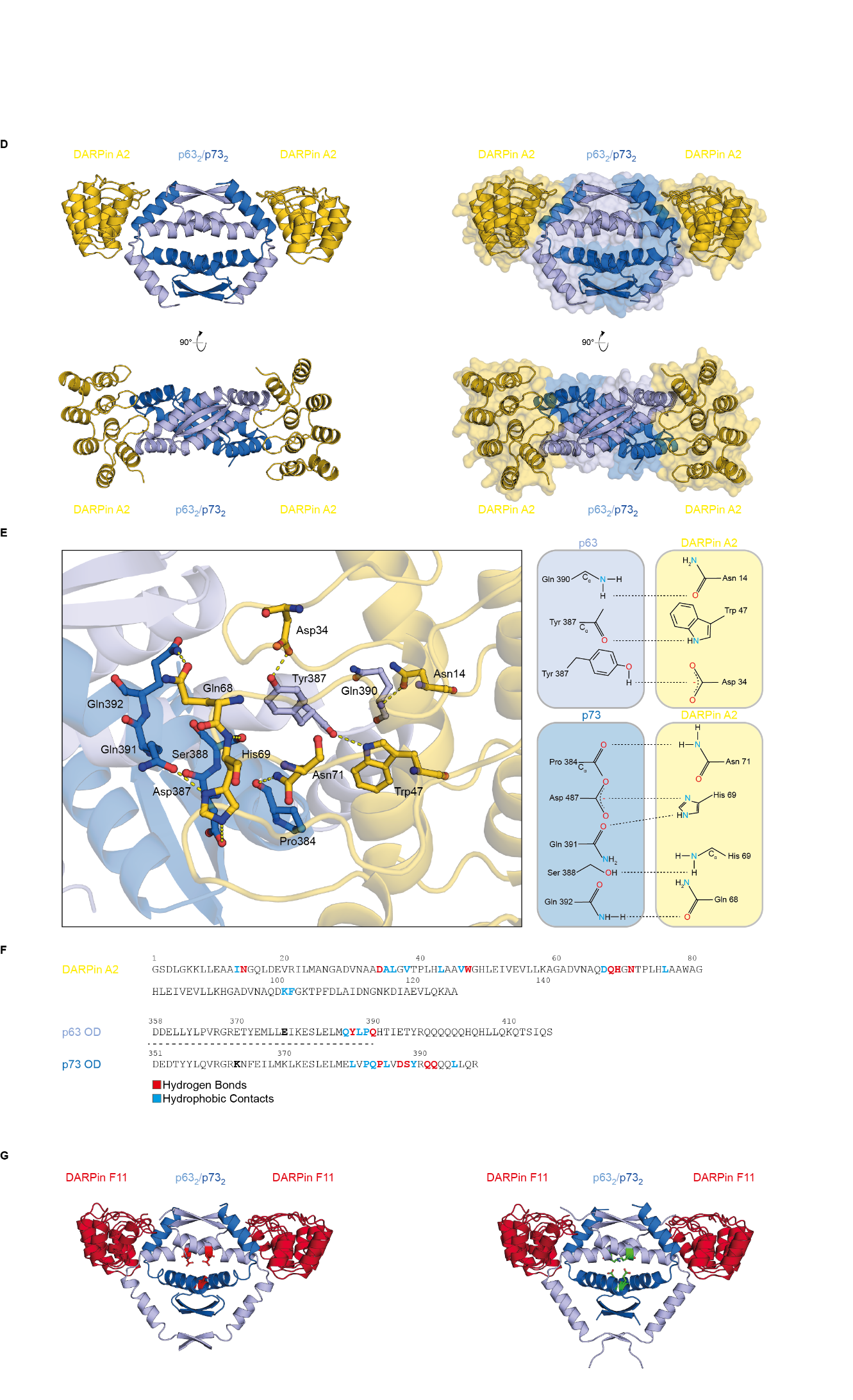


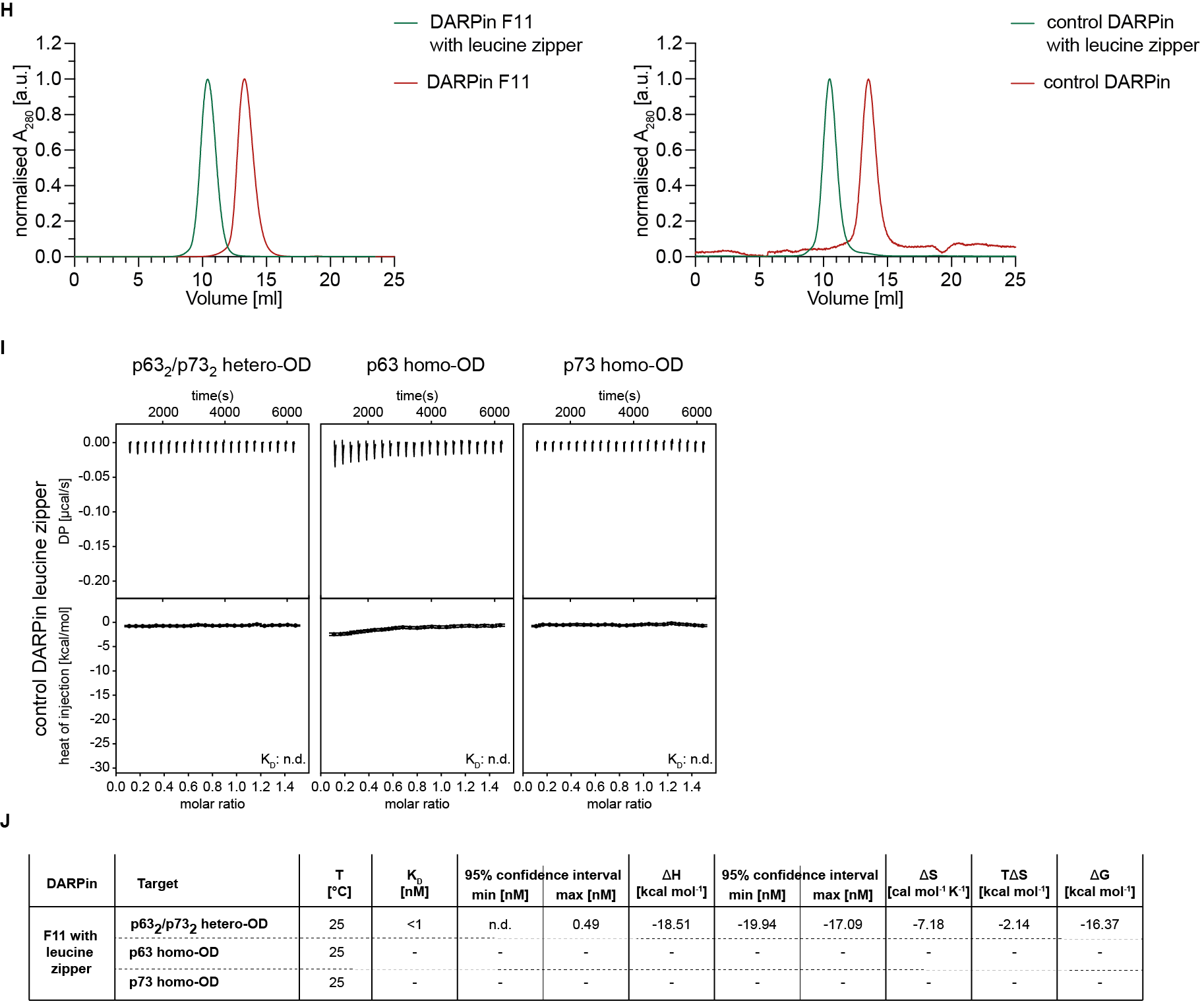


**Supplementary Fig. 3**

(**A**) Size exclusion chromatography of the stabilized p63_2_/p73_2_ hetero-tetramer in comparison with the p63 homo-OD or the p73 homo-OD on a Superdex 75 10/300 column. (**B**) Size exclusion chromatography of DARPins F11 and A2 on a Superdex 75 10/300 column demonstrating that they form only monomers. (**C**) Size exclusion chromatography of a 2:1 mixture of the DARPins and the stabilized p63_2_/p73_2_ hetero-tetramer on a Superdex 75 10/300 column prior to crystallization experiments. (**D**) Crystal structure of a complex of the stabilized p63_2_/p73_2_ hetero-tetramer and DARPin A2 in two different orientations rotated by 90^o^. On the left side secondary structure elements are shown, on the right side these elements are overlaid with a space filling model of the complex. (**E**) Detailed analysis of the interface of DARPin A2 with the hetero-tetramer showing the interacting side chains of the complex crystal structure analyzed by LigPlot. (**F**) Sequence of the DARPin A2 and alignment of OD sequences of p63 and p73 with amino acids highlighted involved in the interaction with the DARPin. Hydrogen bond contacts are highlighted in red, hydrophobic contacts in blue. The two mutations, E363K in the p73 OD and K377E in the p63 OD, that were introduced to stabilize the p63_2_/p73_2_ hetero-tetramer are marked in bold. (**G**) Comparison of the crystal structure of the DARPin F11 in complex with the p73 E363K and p63 K377E mutant (left) with the structure of DARPin F11 in complex with the wild-type p63_2_/p73_2_ hetero-tetramer complex (right). Mutated residues are shown in red in the structure on the left and the wild-type residues in green in the structure on the right. (**H**) Size exclusion chromatography of the monomeric and via leucine zipper dimerized DARPin F11 and control DARPin. (**I**) Results of ITC experiments with the control DARPin dimerized via a leucine zipper. This dimerized control DARPin does not interact with the stabilized p63_2_/p73_2_ hetero-tetramer, the p63 homo-OD or the p73 homo-OD. The top diagram displays the raw measurement and the bottom diagram shows the integrated heat per titration step. (**J**) Summary of the thermodynamic parameters of the interaction of the dimerized DARPin F11 with the stabilized p63_2_/p73_2_ hetero-tetramer, the p63 homo-OD and the p73 homo-OD obtained by ITC measurements.


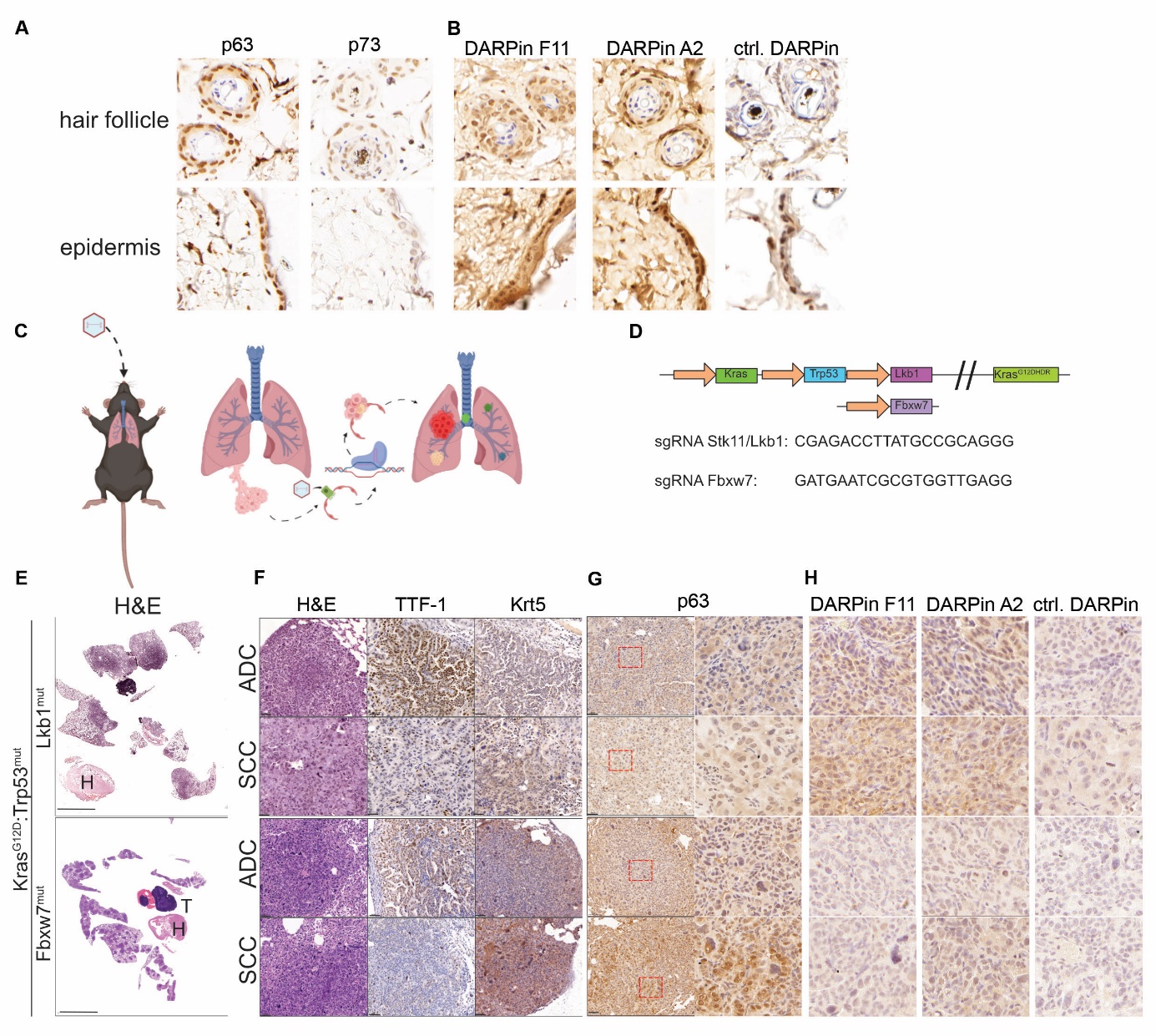


**Supplementary Fig. 4**

DARPins A2 and F11 can be employed to identify squamous lung tumors in murine NSCLC models. (**A**) Immunohistology staining of murine skin sections of endogenous p63 and p73. Shown are representative images of hair follicles and epidermis. Scale bar = 50 µM. (**B**) Immunohistology staining of murine skin sections against the p63_2_/p73_2_ hetero-tetramer using DARPins F11 and A2 as well as a control DARPin. Scale bar = 50 µM. (**C**) Schematic representation of the employed autochthonous CRISPR NSCLC in vivo model. C57Bl6/J Rosa26-CAGG-Cas9-IRES-eGFP animals were intratracheally administered with 50 µl PBS containing AAV encoding sgRNA and HDR templates for the induction of oncogenic transformation in otherwise wild-type lung epithelium. Infected animals were terminated 12 weeks post i.t. (**D**) Schematic overview of the AAV sgRNA and HDR structure used to initiate NSCLC, adenocarcinoma (ADC) and squamous cell carcinoma (SCC) in vivo. sgRNA sequences targeting Stk11/Lkb1 and Fbxw7 are indicated. (**E**) Representative hematoxylin & eosine (H&E) stainings of tumor bearing animals at endpoint after intratracheal infection with AAV targeting Kras, Trp53 and Lkb1 (KPL) or Fbxw7 (KPF), respectively. H = heart, T = thymus. Scale bar = 5 mm. (**F**) Hematoxylin & eosine (H&E) and immunohistology staining of tumors in KPL and KPF mice against the ADC marker TTF-1 (Nkx2-1) and the SCC marker Krt5. ADC = TTF-1+/Krt5-, SCC= TTF-1-/Krt5+. Scale bar = 50 µm. (**G**) Immunohistology staining of ADC and SCC tumors in KPL and KPF mice against TP63. Scale bar = 50 µm. Box indicates area of higher magnification. (**H**) Immunohistology staining of ADC and SCC tumors in KPL and KPF mice against the HA tagged DARPins A2 and F11. Scale bar = 50 µm. Box indicates area of higher magnification.

| **Complex** | YY02XX01TP73A  F11 + p63_2_/p73_2_ hetero-tetramer | YY03XX01TP73A  A2 + p63_2_/p73_2_ hetero-tetramer | YY04XX01TP73A  F11 + wild type p63_2_/p73_2_ hetero-tetramer |
| --- | --- | --- | --- |
| **PDB accession code** | **8P9C** | **8P9D** | **8P9E** |
| ***Data Collection*** |  |  |  |
| Resolution^a^ (Å) | 48.73-1.76 (1.82-1.76) | 45.08-2.70 (2.85-2.70) | 49.05-2.25 (2.33-2.25) |
| Spacegroup | *C* 2 | *P* 2_1_2_1_2_1_ | *C* 2 |
| Cell dimensions | *a* = 84.0, *b* = 60.3, *c* = 53.6 Å | *a* = 40.7, *b* = 113.2, *c* = 135.2 Å | *a* = 84.7, *b* = 60.7, *c* = 53.9 Å |
|  | *α* = *γ* = 90.0*, β* = 100.3° | *α* = *β* = *γ* = 90.0° | *α* = *γ* = 90.0*, β* = 100.8° |
| No. unique reflections^a^ | 25,297 (2,481) | 17,853 (2,555) | 12,746 (1,208) |
| Completeness^a^ (%) | 96.7 (97.0) | 99.5 (100.0) | 99.4 (99.0) |
| I/σI^a^ | 12.2 (2.1) | 11.8 (2.0) | 8.6 (2.1) |
| R_merge_^a^ | 0.049 (0.392) | 0.083 (0.848) | 0.099 (0.777) |
| CC (1/2) | 0.997 (0.899) | 0.998 (0.751) | 0.998 (0.883) |
| Redundancy^a^ | 4.0 (4.1) | 6.0 (6.2) | 7.0 (6.8) |
| ***Refinement*** |  |  |  |
| No. atoms in refinement (P/O)^b^ | 2,086/ 180 | 3,458/ 6 | 2,068/ 35 |
| B factor (P/O)^b^ (Å^2^) | 34/ 40 | 83/ 58 | 62/ 61 |
| R_fact_ (%) | 17.0 | 21.5 | 18.9 |
| R_free_ (%) | 21.0 | 25.2 | 25.0 |
| rms deviation bond^c^ (Å) | 0.016 | 0.009 | 0.011 |
| rms deviation angle^c^ (°) | 1.5 | 1.1 | 1.3 |
| Crystallization conditions | 0.2M ammonium acetate, 25% PEG3350, 0.1M HEPES pH 7.5 | 20% PEG3350, 0.1M bis-tris propane pH 7.0, 0.2M salicylic, sodium salt | 0.2M sodium chloride, 25% PEG3350, 0.1M bis-tris pH 6.5 |

**Supplementary Table 1** crystallographic statistics

^a^ Values in brackets show the statistics for the highest resolution shells.

^b^ P/O indicate protein and others (water and solvent molecules), respectively.

^c^ rms indicates root-mean-square.
