# Supplementary material for: DARPins detect the formation of hetero-tetramers of p63 and p73 in epithelial tissues and in squamous cell carcinoma
Source: Cell Death Dis. 2023 Oct 12;14(10):674. doi: 10.1038/s41419-023-06213-0 (PMC10570377; doi:10.1038/s41419-023-06213-0)
Supplement: Supplementary file 2 — Original Data File [file 41419_2023_6213_MOESM2_ESM.pdf]

Raw data figure 2c

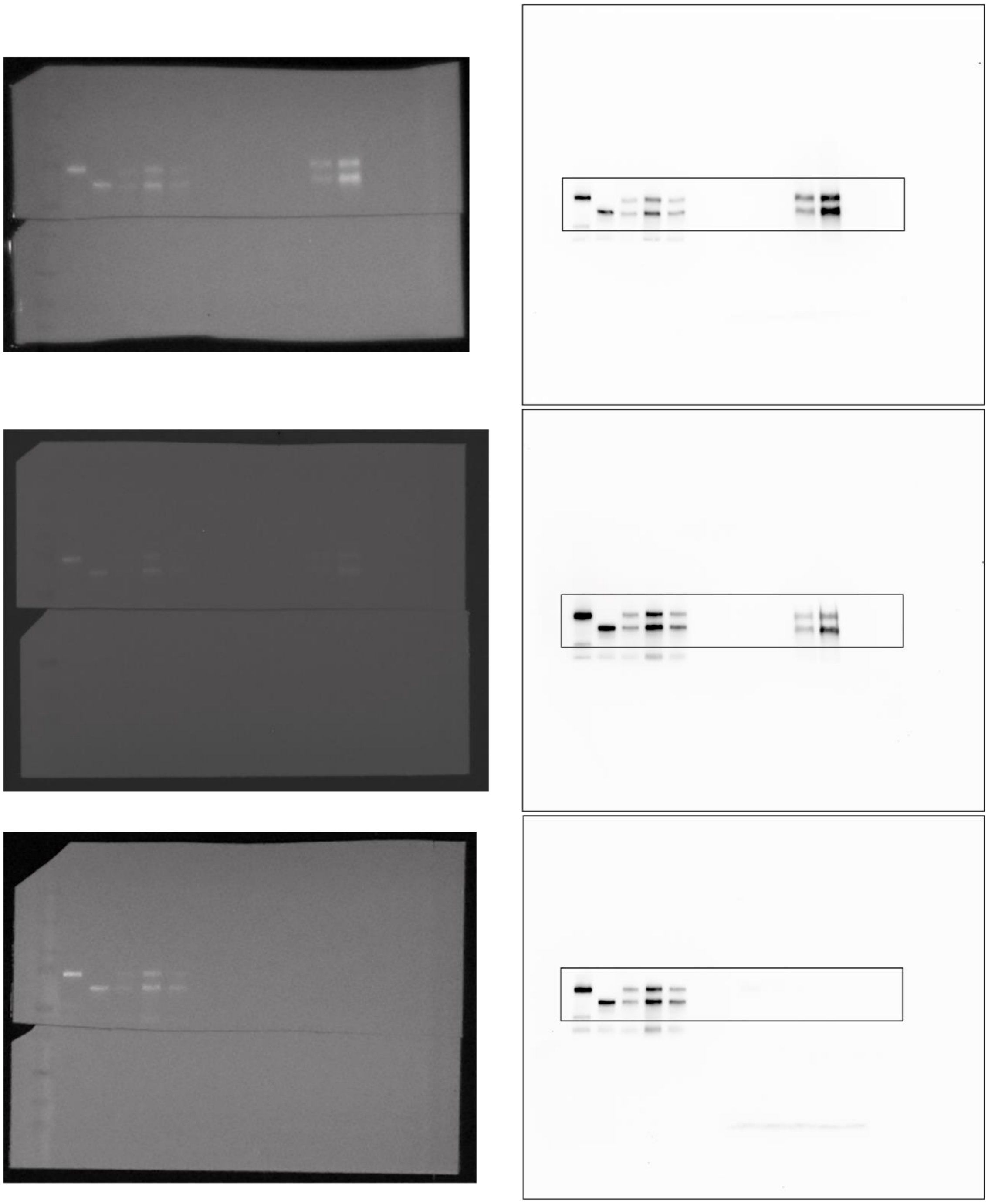

Uncropped Western blots used in Figure 2C. The boxes indicate the area shown in figure.

Raw data figure 2d

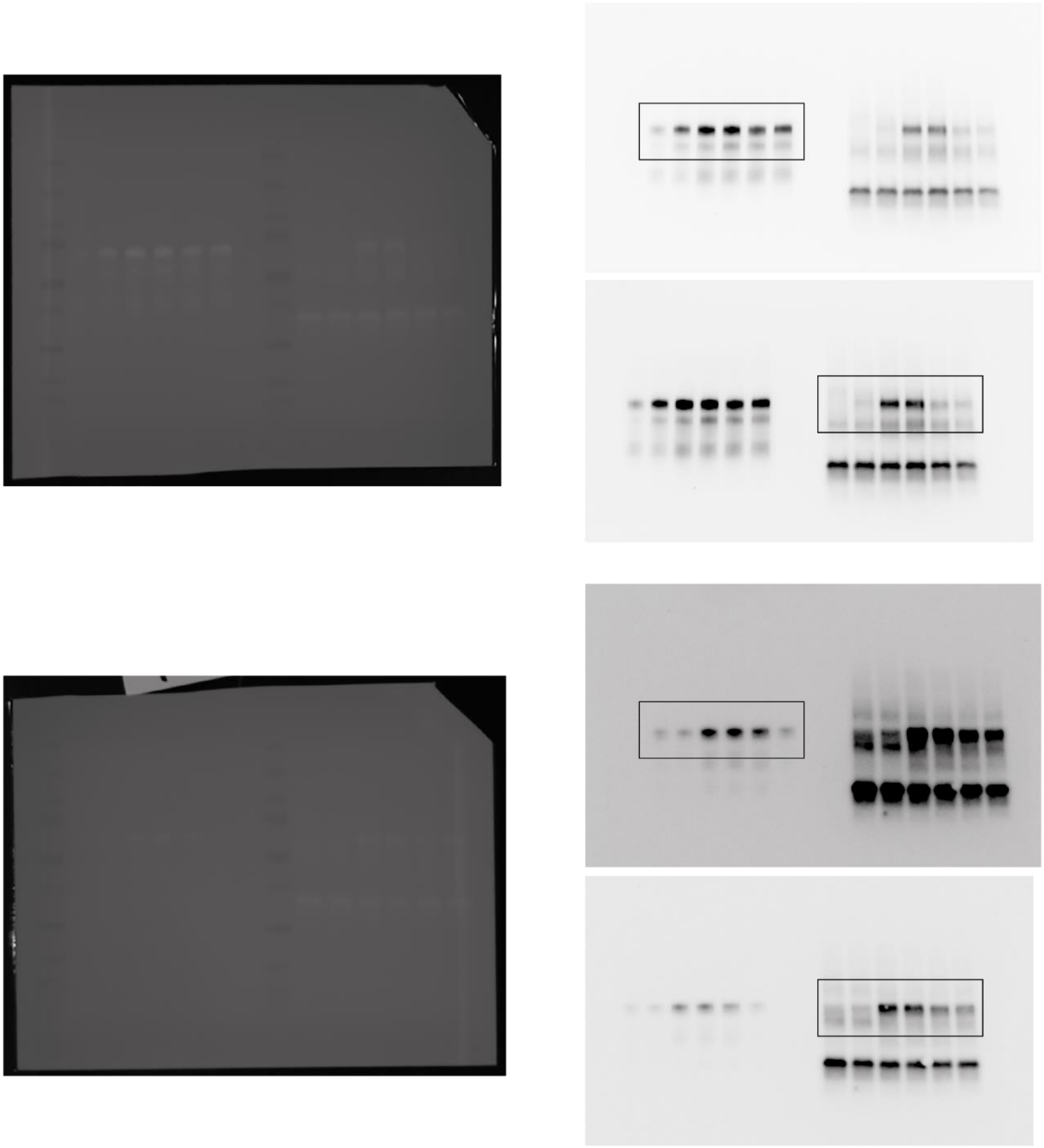

Uncropped Western blots used in Figure 2d. The boxes indicate the area shown in figure.

Raw data figure 5

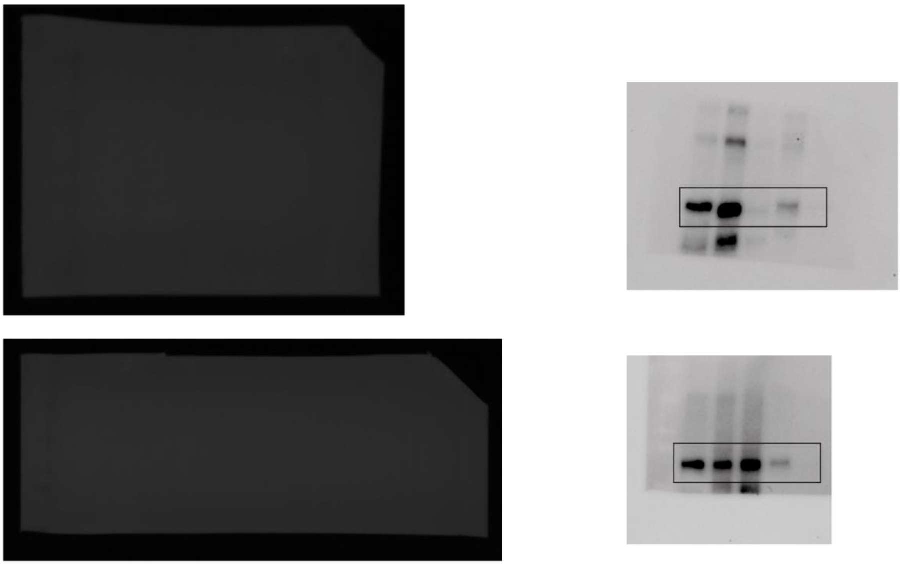

Uncropped Western blots used in Figure 5. The boxes indicate the area shown in figure.
