# Supplementary material for: DARPins detect the formation of hetero-tetramers of p63 and p73 in epithelial tissues and in squamous cell carcinoma
Source: Cell Death Dis. 2023 Oct 12;14(10):674. doi: 10.1038/s41419-023-06213-0 (PMC10570377; doi:10.1038/s41419-023-06213-0)
Supplement: Supplementary file 5 — 8P9D validation report [file 41419_2023_6213_MOESM5_ESM.pdf]

Full wwPDB X-ray Structure Validation Report (i)

Jun 6, 2023 – 11:30 am BST

PDB ID	:	8P9D
Title	:	Crystal structure of p63-p73 heterotetramer (tetramerisation domain) in complex with darpin 1810 A2
Deposited on	:	2023-06-05
Resolution	:	2.70 Å(reported)

This wwPDB validation report is for manuscript review

This is a Full wwPDB X-ray Structure Validation Report.

This report is produced by the wwPDB biocuration pipeline after annotation of the structure.

We welcome your comments at *validation@mail.wwpdb.org*
A user guide is available at
https://www.wwpdb.org/validation/2017/XrayValidationReportHelp
with specific help available everywhere you see the (i) symbol.

The types of validation reports are described at
http://www.wwpdb.org/validation/2017/FAQs#types.

The following versions of software and data (see references (i)) were used in the production of this report:

MolProbity	:	4.02b-467
Xtriage (Phenix)	:	1.13
EDS	:	2.33
Percentile statistics	:	20191225.v01 (using entries in the PDB archive December 25th 2019)
Refmac	:	5.8.0158
CCP4	:	7.0.044 (Gargrove)
Ideal geometry (proteins)	:	Engh & Huber (2001)
Ideal geometry (DNA, RNA)	:	Parkinson et al. (1996)
Validation Pipeline (wwPDB-VP)	:	2.33

For Manuscript Review

1 Overall quality at a glance (i)

The following experimental techniques were used to determine the structure:
X-RAY DIFFRACTION

The reported resolution of this entry is 2.70 Å.

Percentile scores (ranging between 0-100) for global validation metrics of the entry are shown in the following graphic. The table shows the number of entries on which the scores are based.

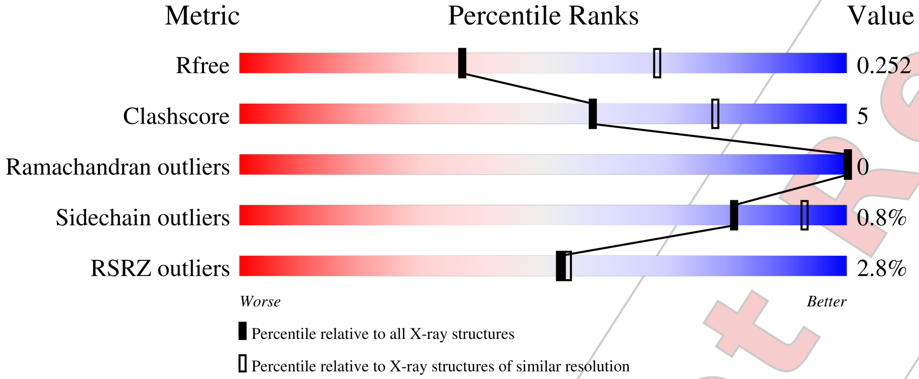

Metric	Whole archive (#Entries)	Similar resolution (#Entries, resolution range(Å))
R_{free}	130704	2808 (2.70-2.70)
Clashscore	141614	3122 (2.70-2.70)
Ramachandran outliers	138981	3069 (2.70-2.70)
Sidechain outliers	138945	3069 (2.70-2.70)
RSRZ outliers	127900	2737 (2.70-2.70)

The table below summarises the geometric issues observed across the polymeric chains and their fit to the electron density. The red, orange, yellow and green segments of the lower bar indicate the fraction of residues that contain outliers for >=3, 2, 1 and 0 types of geometric quality criteria respectively. A grey segment represents the fraction of residues that are not modelled. The numeric value for each fraction is indicated below the corresponding segment, with a dot representing fractions <=5% The upper red bar (where present) indicates the fraction of residues that have poor fit to the electron density. The numeric value is given above the bar.

Mol	Chain	Length	Quality of chain
1	A	61	2% / 64% 11% 25%
1	C	61	8% / 62% 13% 25%
2	B	50	8% / 72% 24% •
2	D	50	4% / 74% 18% 8%

Continued on next page...

For Manuscript Review

Continued from previous page...

Mol	Chain	Length	Quality of chain
3	E	126	90% 10% •
3	F	126	90% 10% •

For Manuscript Review

2 Entry composition (i)

There are 4 unique types of molecules in this entry. The entry contains 3464 atoms, of which 0 are hydrogens and 0 are deuteriums.

In the tables below, the ZeroOcc column contains the number of atoms modelled with zero occupancy, the AltConf column contains the number of residues with at least one atom in alternate conformation and the Trace column contains the number of residues modelled with at most 2 atoms.

- Molecule 1 is a protein called Tumor protein 63.

Mol	Chain	Residues	Atoms					ZeroOcc	AltConf	Trace
1	A	46	Total 401	C 253	N 68	O 78	S 2	0	0	0
1	C	46	Total 401	C 253	N 68	O 78	S 2	0	0	0

There are 6 discrepancies between the modelled and reference sequences:

Chain	Residue	Modelled	Actual	Comment	Reference
A	356	GLY	-	expression tag	UNP Q9H3D4
A	357	SER	-	expression tag	UNP Q9H3D4
A	377	GLU	LYS	conflict	UNP Q9H3D4
C	356	GLY	-	expression tag	UNP Q9H3D4
C	357	SER	-	expression tag	UNP Q9H3D4
C	377	GLU	LYS	conflict	UNP Q9H3D4

- Molecule 2 is a protein called Tumor protein p73.

Mol	Chain	Residues	Atoms					ZeroOcc	AltConf	Trace
2	B	48	Total 407	C 258	N 68	O 79	S 2	0	0	0
2	D	46	Total 393	C 251	N 66	O 74	S 2	0	0	0

There are 6 discrepancies between the modelled and reference sequences:

Chain	Residue	Modelled	Actual	Comment	Reference
B	349	GLY	-	expression tag	UNP O15350
B	350	SER	-	expression tag	UNP O15350
B	363	LYS	GLU	conflict	UNP O15350
D	349	GLY	-	expression tag	UNP O15350
D	350	SER	-	expression tag	UNP O15350
D	363	LYS	GLU	conflict	UNP O15350

For Manuscript Review

- Molecule 3 is a protein called darpin 1810 A2.

Mol	Chain	Residues	Atoms					ZeroOcc	AltConf	Trace
3	E	125	Total 928	C 585	N 163	O 179	S 1	0	0	0
3	F	125	Total 928	C 585	N 163	O 179	S 1	0	0	0

- Molecule 4 is water.

Mol	Chain	Residues	Atoms		ZeroOcc	AltConf
4	A	1	Total 1	O 1	0	0
4	C	1	Total 1	O 1	0	0
4	E	2	Total 2	O 2	0	0
4	F	2	Total 2	O 2	0	0

For Manuscript Review

3 Residue-property plots (i)

These plots are drawn for all protein, RNA, DNA and oligosaccharide chains in the entry. The first graphic for a chain summarises the proportions of the various outlier classes displayed in the second graphic. The second graphic shows the sequence view annotated by issues in geometry and electron density. Residues are color-coded according to the number of geometric quality criteria for which they contain at least one outlier: green = 0, yellow = 1, orange = 2 and red = 3 or more. A red dot above a residue indicates a poor fit to the electron density (RSRZ > 2). Stretches of 2 or more consecutive residues without any outlier are shown as a green connector. Residues present in the sample, but not in the model, are shown in grey.

- Molecule 1: Tumor protein 63

Chain A:

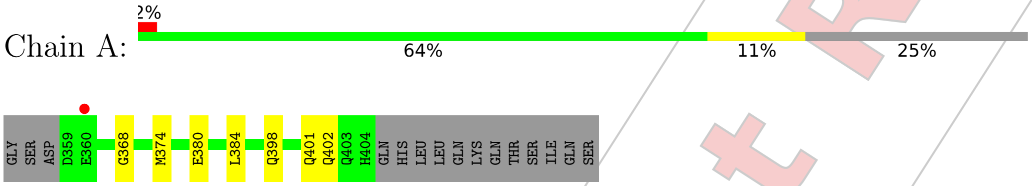

- Molecule 1: Tumor protein 63

Chain C:

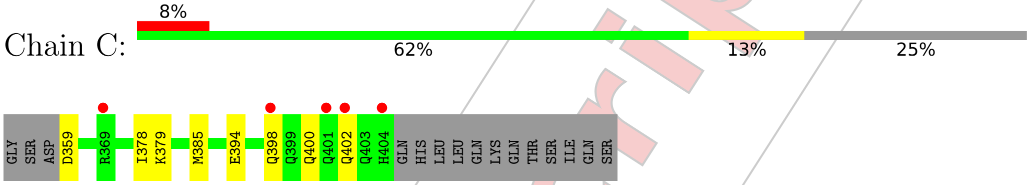

- Molecule 2: Tumor protein p73

Chain B:

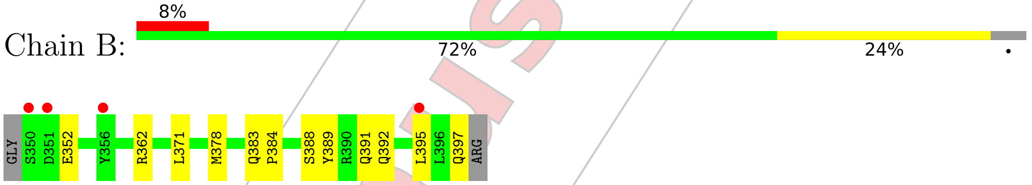

- Molecule 2: Tumor protein p73

Chain D:

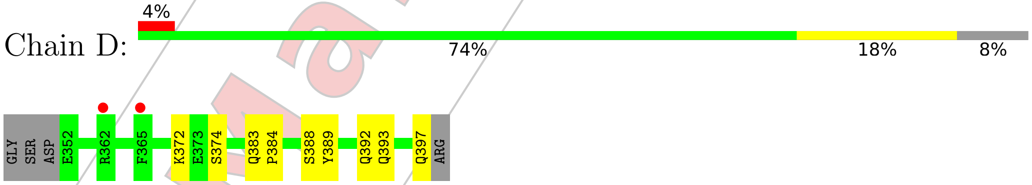

- Molecule 3: darpin 1810 A2

Chain E:

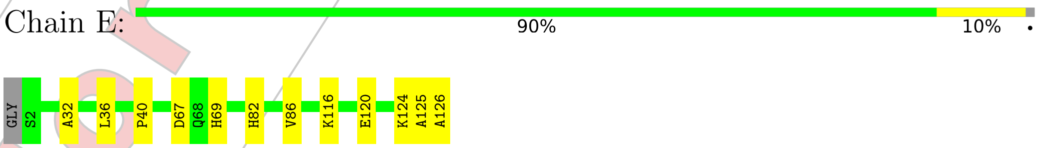

- Molecule 3: darpin 1810 A2

For Manuscript Review

Chain F:

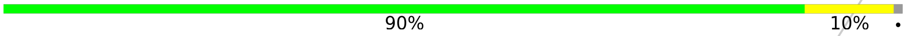

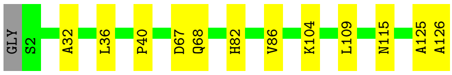

For Manuscript Review

4 Data and refinement statistics (i)

Property	Value	Source
Space group	P 21 21 21	Depositor
Cell constants<br>a, b, c, α, β, γ	40.73Å 113.19Å 135.23Å<br>90.00° 90.00° 90.00°	Depositor
Resolution (Å)	43.40 – 2.70<br>43.40 – 2.70	Depositor<br>EDS
% Data completeness<br>(in resolution range)	99.3 (43.40-2.70)<br>99.4 (43.40-2.70)	Depositor<br>EDS
R_{merge}	0.08	Depositor
R_{sym}	(Not available)	Depositor
$< I/\sigma(I) >$ [1]	2.19 (at 2.69Å)	Xtriage
Refinement program	REFMAC 5.8.0238	Depositor
R, R_{free}	0.215 , 0.252<br>0.216 , 0.252	Depositor<br>DCC
R_{free} test set	906 reflections (5.09%)	wwPDB-VP
Wilson B-factor (Å^2)	66.0	Xtriage
Anisotropy	0.588	Xtriage
Bulk solvent k_{sol}(e/Å^3), B_{sol}(Å^2)	0.33 , 45.0	EDS
L-test for twinning[2]	$<\|L\|> = 0.48$, $< L^2 > = 0.31$	Xtriage
Estimated twinning fraction	No twinning to report.	Xtriage
F_o,F_c correlation	0.95	EDS
Total number of atoms	3464	wwPDB-VP
Average B, all atoms (Å^2)	82.0	wwPDB-VP

Xtriage's analysis on translational NCS is as follows: *The largest off-origin peak in the Patterson function is 6.13% of the height of the origin peak. No significant pseudotranslation is detected.*

[1]Intensities estimated from amplitudes.

[2]Theoretical values of $<|L|>$, $< L^2 >$ for acentric reflections are 0.5, 0.333 respectively for untwinned datasets, and 0.375, 0.2 for perfectly twinned datasets.

For Manuscript Review

5 Model quality (i)

5.1 Standard geometry (i)

The Z score for a bond length (or angle) is the number of standard deviations the observed value is removed from the expected value. A bond length (or angle) with $|Z| > 5$ is considered an outlier worth inspection. RMSZ is the root-mean-square of all Z scores of the bond lengths (or angles).

Mol	Chain	Bond lengths		Bond angles	
		RMSZ	#|Z| >5	RMSZ	#|Z| >5
1	A	0.68	0/408	0.66	0/550
1	C	0.74	0/408	0.65	0/550
2	B	0.67	0/412	0.64	0/553
2	D	0.66	0/398	0.63	0/534
3	E	0.72	0/942	0.70	0/1280
3	F	0.74	0/942	0.69	0/1280
All	All	0.71	0/3510	0.67	0/4747

There are no bond length outliers.

There are no bond angle outliers.

There are no chirality outliers.

There are no planarity outliers.

5.2 Too-close contacts (i)

In the following table, the Non-H and H(model) columns list the number of non-hydrogen atoms and hydrogen atoms in the chain respectively. The H(added) column lists the number of hydrogen atoms added and optimized by MolProbity. The Clashes column lists the number of clashes within the asymmetric unit, whereas Symm-Clashes lists symmetry-related clashes.

Mol	Chain	Non-H	H(model)	H(added)	Clashes	Symm-Clashes
1	A	401	0	391	5	0
1	C	401	0	391	8	0
2	B	407	0	411	9	0
2	D	393	0	402	6	0
3	E	928	0	927	7	1
3	F	928	0	927	7	1
4	A	1	0	0	0	0
4	C	1	0	0	0	0
4	E	2	0	0	0	0
4	F	2	0	0	0	0

Continued on next page...

For Manuscript Review

Continued from previous page...

Mol	Chain	Non-H	H(model)	H(added)	Clashes	Symm-Clashes
All	All	3464	0	3449	33	1

The all-atom clashscore is defined as the number of clashes found per 1000 atoms (including hydrogen atoms). The all-atom clashscore for this structure is 5.

All (33) close contacts within the same asymmetric unit are listed below, sorted by their clash magnitude.

Atom-1	Atom-2	Interatomic distance (Å)	Clash overlap (Å)
2:B:352:GLU:HA	2:B:352:GLU:OE1	1.97	0.65
2:B:388:SER:HA	3:E:69:HIS:HB2	1.80	0.63
3:E:125:ALA:O	3:E:126:ALA:OXT	2.21	0.59
1:C:398:GLN:O	1:C:402:GLN:HG2	2.03	0.58
3:F:125:ALA:O	3:F:126:ALA:OXT	2.22	0.57
2:B:391:GLN:O	2:B:395:LEU:HG	2.04	0.56
2:D:393:GLN:O	2:D:397:GLN:HG3	2.08	0.53
3:E:120:GLU:O	3:E:124:LYS:HG3	2.08	0.53
1:C:379:LYS:HE2	2:D:389:TYR:OH	2.09	0.52
1:A:380:GLU:HG3	2:B:389:TYR:CE2	2.45	0.51
1:C:398:GLN:O	1:C:402:GLN:NE2	2.40	0.49
2:B:371:LEU:HD11	1:C:378:ILE:HD11	1.94	0.49
2:D:383:GLN:HB3	2:D:384:PRO:HD3	1.94	0.49
2:D:392:GLN:NE2	3:F:68:GLN:OE1	2.45	0.49
3:E:32:ALA:O	3:E:40:PRO:HD3	2.14	0.48
3:F:104:LYS:HB3	3:F:109:LEU:CD1	2.43	0.47
2:B:383:GLN:HB3	2:B:384:PRO:HD3	1.98	0.45
1:C:394:GLU:HA	1:C:394:GLU:OE2	2.17	0.45
2:B:392:GLN:HA	2:B:395:LEU:HB2	2.00	0.44
3:F:82:HIS:O	3:F:86:VAL:HG23	2.17	0.44
2:B:397:GLN:O	2:B:397:GLN:HG3	2.18	0.43
1:A:368:GLY:HA2	1:C:359:ASP:HA	2.01	0.42
3:F:36:LEU:O	3:F:67:ASP:HB2	2.20	0.42
1:C:400:GLN:CD	2:D:372:LYS:HZ2	2.23	0.42
3:F:32:ALA:O	3:F:40:PRO:HD3	2.20	0.41
1:A:398:GLN:HA	1:A:401:GLN:HG2	2.02	0.41
3:E:82:HIS:O	3:E:86:VAL:HG23	2.20	0.41
3:F:125:ALA:O	3:F:126:ALA:C	2.58	0.41
3:E:125:ALA:O	3:E:126:ALA:C	2.58	0.41
1:C:385:MET:SD	2:D:374:SER:HA	2.60	0.41
3:E:36:LEU:O	3:E:67:ASP:HB2	2.22	0.40
1:A:384:LEU:HD12	2:B:378:MET:CE	2.51	0.40
1:A:398:GLN:O	1:A:402:GLN:HG2	2.21	0.40

For Manuscript Review

All (1) symmetry-related close contacts are listed below. The label for Atom-2 includes the symmetry operator and encoded unit-cell translations to be applied.

Atom-1	Atom-2	Interatomic distance (Å)	Clash overlap (Å)
3:E:116:LYS:NZ	3:F:115:ASN:OD1[2_554]	2.15	0.05

5.3 Torsion angles (i)

5.3.1 Protein backbone (i)

In the following table, the Percentiles column shows the percent Ramachandran outliers of the chain as a percentile score with respect to all X-ray entries followed by that with respect to entries of similar resolution.

The Analysed column shows the number of residues for which the backbone conformation was analysed, and the total number of residues.

Mol	Chain	Analysed	Favoured	Allowed	Outliers	Percentiles	
1	A	44/61 (72%)	44 (100%)	0	0	100	100
1	C	44/61 (72%)	44 (100%)	0	0	100	100
2	B	46/50 (92%)	42 (91%)	4 (9%)	0	100	100
2	D	44/50 (88%)	41 (93%)	3 (7%)	0	100	100
3	E	123/126 (98%)	119 (97%)	4 (3%)	0	100	100
3	F	123/126 (98%)	119 (97%)	4 (3%)	0	100	100
All	All	424/474 (90%)	409 (96%)	15 (4%)	0	100	100

There are no Ramachandran outliers to report.

5.3.2 Protein sidechains (i)

In the following table, the Percentiles column shows the percent sidechain outliers of the chain as a percentile score with respect to all X-ray entries followed by that with respect to entries of similar resolution.

The Analysed column shows the number of residues for which the sidechain conformation was analysed, and the total number of residues.

Mol	Chain	Analysed	Rotameric	Outliers	Percentiles	
1	A	45/59 (76%)	44 (98%)	1 (2%)	52	79
1	C	45/59 (76%)	45 (100%)	0	100	100

Continued on next page...

For Manuscript Review

Continued from previous page...

Mol	Chain	Analysed	Rotameric	Outliers	Percentiles	
2	B	47/48 (98%)	46 (98%)	1 (2%)	53	80
2	D	45/48 (94%)	44 (98%)	1 (2%)	52	79
3	E	93/93 (100%)	93 (100%)	0	100	100
3	F	93/93 (100%)	93 (100%)	0	100	100
All	All	368/400 (92%)	365 (99%)	3 (1%)	81	93

All (3) residues with a non-rotameric sidechain are listed below:

Mol	Chain	Res	Type
1	A	374	MET
2	B	362	ARG
2	D	388	SER

Sometimes sidechains can be flipped to improve hydrogen bonding and reduce clashes. There are no such sidechains identified.

5.3.3 RNA ⓘ

There are no RNA molecules in this entry.

5.4 Non-standard residues in protein, DNA, RNA chains ⓘ

There are no non-standard protein/DNA/RNA residues in this entry.

5.5 Carbohydrates ⓘ

There are no monosaccharides in this entry.

5.6 Ligand geometry ⓘ

There are no ligands in this entry.

5.7 Other polymers ⓘ

There are no such residues in this entry.

For Manuscript Review

5.8 Polymer linkage issues (i)

There are no chain breaks in this entry.

For Manuscript Review

6 Fit of model and data (i)

6.1 Protein, DNA and RNA chains (i)

In the following table, the column labelled '#RSRZ> 2' contains the number (and percentage) of RSRZ outliers, followed by percent RSRZ outliers for the chain as percentile scores relative to all X-ray entries and entries of similar resolution. The OWAB column contains the minimum, median, 95th percentile and maximum values of the occupancy-weighted average B-factor per residue. The column labelled 'Q< 0.9' lists the number of (and percentage) of residues with an average occupancy less than 0.9.

Mol	Chain	Analysed	<RSRZ>	#RSRZ>2			OWAB(Å^2)	Q<0.9
1	A	46/61 (75%)	0.30	1 (2%)	62	63	59, 86, 132, 150	0
1	C	46/61 (75%)	0.68	5 (10%)	5	4	58, 89, 149, 162	0
2	B	48/50 (96%)	0.63	4 (8%)	11	9	58, 83, 153, 163	0
2	D	46/50 (92%)	0.43	2 (4%)	35	33	53, 81, 118, 128	0
3	E	125/126 (99%)	-0.11	0	100	100	51, 69, 92, 116	0
3	F	125/126 (99%)	0.08	0	100	100	54, 74, 102, 132	0
All	All	436/474 (91%)	0.21	12 (2%)	53	54	51, 76, 126, 163	0

All (12) RSRZ outliers are listed below:

Mol	Chain	Res	Type	RSRZ
2	B	350	SER	4.8
1	C	402	GLN	4.1
2	B	395	LEU	3.7
1	C	401	GLN	3.7
1	C	404	HIS	3.5
2	B	351	ASP	3.4
1	C	369	ARG	2.8
2	B	356	TYR	2.7
2	D	365	PHE	2.6
1	A	360	GLU	2.5
2	D	362	ARG	2.5
1	C	398	GLN	2.2

6.2 Non-standard residues in protein, DNA, RNA chains (i)

There are no non-standard protein/DNA/RNA residues in this entry.

For Manuscript Review

6.3 Carbohydrates (i)

There are no monosaccharides in this entry.

6.4 Ligands (i)

There are no ligands in this entry.

6.5 Other polymers (i)

There are no such residues in this entry.

For Manuscript Review
